# Refining muscle geometry and wrapping in the TLEM 2 model for improved hip contact force prediction

**DOI:** 10.1371/journal.pone.0204109

**Published:** 2018-09-17

**Authors:** Enrico De Pieri, Morten E. Lund, Anantharaman Gopalakrishnan, Kasper P. Rasmussen, David E. Lunn, Stephen J. Ferguson

**Affiliations:** 1 Institute for Biomechanics, ETH Zurich, Zurich, Switzerland; 2 AnyBody Technology A/S, Aalborg, Denmark; 3 Leeds Teaching Hospitals National Health Service Trust, Leeds, United Kingdom; University of Guelph, CANADA

## Abstract

Musculoskeletal models represent a powerful tool to gain knowledge on the internal forces acting at the joint level in a non-invasive way. However, these models can present some errors associated with the level of detail in their geometrical representation. For this reason, a thorough validation is necessary to prove the reliability of their predictions. This study documents the development of a generic musculoskeletal model and proposes a working logic and simulation techniques for identifying specific model features in need of refinement; as well as providing a quantitative validation for the prediction of hip contact forces (HCF). The model, implemented in the AnyBody Modeling System and based on the cadaveric dataset TLEM 2.0, was scaled to match the anthropometry of a patient fitted with an instrumented hip implant and to reproduce gait kinematics based on motion capture data. The relative contribution of individual muscle elements to the HCF and joint moments was analyzed to identify critical geometries, which were then compared to muscle magnetic resonance imaging (MRI) scans and, in case of inconsistencies, were modified to better match the volumetric scans. The predicted HCF showed good agreement with the overall trend and timing of the measured HCF from the instrumented prosthesis. The average root mean square error (RMSE), calculated for the total HCF was found to be 0.298*BW. Refining the geometries of the muscles thus identified reduced RMSE on HCF magnitudes by 17% (from 0.359*BW to 0.298*BW) over the whole gait cycle. The detailed study of individual muscle contributions to the HCF succeeded in identifying muscles with incorrect anatomy, which would have been difficult to intuitively identify otherwise. Despite a certain residual over-prediction of the final hip contact forces in the stance phase, a satisfactory level of geometrical accuracy of muscle paths has been achieved with the refinement of this model.

## Introduction

Accurate knowledge of the internal forces acting at the joint level is necessary for the improvement of total joint replacement designs and for defining more realistic pre-clinical testing [1,2]. Lubrication and wear behavior of the implant [3,4], as well as stress distribution in the periprosthetic bone [5], are particularly affected by the loading conditions, patients’ activity and anatomy [6]. In-vivo measurements have been previously obtained from patients with the aid of instrumented prostheses for hip [7,8] and knee [9,10], but further data acquisition is limited due to ethical and practical considerations. Additionally, these measurements only reflect a small sample of the population, therefore they do not accurately represent real world loading scenarios.

Musculoskeletal models [11,12] have the potential to overcome this limitation and can provide estimates for internal joint and muscle forces derived from kinematic and kinetic data acquired from subjects in a motion-capture lab. Through inverse dynamics calculations, these models can compute the necessary joint moments to perform the given kinematic task. The redundancy of the musculoskeletal system is then solved using an optimization algorithm that allows finding an optimal combination of muscle activations and forces that generate the necessary moments [10,13].

An in-silico approach allows to easily investigate the influence of specific clinical factors, such as muscle impairment [14,15] and implant placement [6,16], as well as to expand findings to different target populations and activities [17–19]. These models also allow the estimation of muscle activities and forces, which play a critical role in injury prevention and rehabilitation but cannot be readily measured directly in-vivo otherwise [13]. Despite their potential, musculoskeletal models still need to be thoroughly validated to extend the use of their results to the clinical practice [20,21]. In this sense, a validation of the quantity of interest that is being predicted is necessary, and the range of validity and applicability of these models must be accurately defined [20].

While comparisons between predicted muscle activities and measured electromyography (EMG) signals have previously been used as a qualitative and indirect form of validation [13], other validation options are preferred. Direct and quantitative validation metrics would provide the final user of the model with the confidence to interpret not only trends, but also the magnitudes of the model’s predictions. For this reason, projects like the *Grand Challenge Competition to Predict In Vivo Knee Loads* [10], by providing matching motion capture data, EMG signals, and measured joint contact forces, serve as an important platform to test the performance of musculoskeletal models. In this context, a recent release of one sample dataset of synchronized motion capture and measured hip contact forces [8] from the Orthoload database represents a valuable opportunity to further test the accuracy of newly developed musculoskeletal models with regard to hip contact forces prediction, and to identify major sources of error.

One of the major factors that could affect the prediction of joint and muscle forces is the accuracy of the geometrical representation of the lower-limb muscles [22–26]. In particular, musculoskeletal models were previously reported to be sensitive to errors in the insertion, intermediate, and origin points of the muscles [24,25], with the muscles spanning the hip joint causing the highest uncertainty in the prediction of muscle [25] and contact forces [26]. A recent cadaveric dataset based on medical imaging data, TLEM 2.0 [27], provides muscular geometrical information with the highest level of detail currently available; however, this data has not yet been adopted for musculoskeletal applications focusing on the hip joint.

This study documents the development of a generic lower limb model based on the recent cadaveric dataset TLEM 2.0 [27] and proposes a working logic and simulation techniques for identifying specific model features in need of refinement, with a focus on the definition of muscle insertions and lines of action. The reproduction of hip contact forces from the experiments by Bergmann et al. [8] was chosen as the initial step in the validation process of this newly developed model.

## Material and methods

### Musculoskeletal model

The musculoskeletal model was implemented in the AnyBody Modeling System (v. 7.0.1, AnyBody Technology A/S, Aalborg, Denmark) based on the detailed muscular geometry of the cadaveric dataset TLEM 2.0 [27].

The model consists of a simplified upper body (lumbar region, rigid trunk, neck, and head) and 11 segments representing the lower limbs: pelvis, right and left femurs, patellas, shanks, tali, and feet. Each lower limb comprises four joints: the hip joint is modelled as a 3 degrees of freedom (DOF) ball-and-socket, while knee, talocrural and subtalar joints are modelled as 1-DOF hinges. Additionally, the position of the patella is defined as a function of the knee flexion angle, therefore not introducing additional DOFs.

The model contains 55 muscle actuators in each leg, divided into 169 elements in accordance with the original TLEM dataset [27,28]. Coordinates of insertion and origin points of the single elements were extracted from the contours of measured attachment areas. The muscle elements were modelled with a simple muscle model represented by constant strength actuators.

### Kinematic input, model scaling, and inverse dynamics analysis

This model was linearly scaled to match the anthropometry of a patient fitted with an instrumented hip implant, whose marker coordinates, ground reaction forces (GRF) and hip contact forces (HCFs) measured during a single gait trial have been released online [29].

The patient, H2R, (male, age = 62 years, height = 1.72m, and weight = 78 kg) performed the kinematic trial 12 months after total hip replacement (THR) surgery and his fully anonymized data were accessed from the public repository [29].

The force plate set-up was recreated in the virtual environment following the description included in the dataset. Model markers were also added to the model according to the trunk and lower-body marker protocol available in the dataset. Model marker positions and segment lengths were optimized in a routine that minimized the cumulative error between real and virtual markers during the gait cycle [30,31]. The optimization routine was characterized by a weighting function in which higher penalties were assigned to the markers placed on palpable bony landmarks. The anterior/posterior superior iliac spine (ASIS/PSIS) markers were optimized while constraining the model hip joint position to follow the “Harrington et al.” regression formula [32], which specifies the relationship between the ASIS/PSIS points and the hip joint center. Scaling of model strength and segment sizes (besides segments length) was done based the “Length-Mass-Fat” scaling law described by Rasmussen et al. [33].

The marker and GRF data were first filtered through a second-order zero phase low-pass filter with a cut-off frequency of 5Hz. The optimized kinematics and GRFs then served as input to an inverse dynamic analysis to calculate muscle forces and HCFs. The analysis was based on a 3^rd^ order polynomial muscle recruitment criterion which minimized the sum of muscle activations cubed [13].

### Validation metrics

#### Hip contact forces

The HCFs were derived from the inverse dynamic analysis; a physical interpretation of how the HCF value relates to other forces acting on the body is described further below in the section on “Muscle contribution study and geometry modifications”.

The HCFs computed for the right hip over one gait cycle, were then transformed to a common femur-based reference frame [8] and quantitatively compared to the measurements from the instrumented implant.

The root mean square error (RMSE) was calculated over the whole gait cycle for antero-posterior, medio-lateral, proximo-distal components, as well as for the total HCF, defined as the square root of the sum of the three components squared. RMSE of the total HCF was also evaluated separately for swing and stance phase, to obtain a more localized description of the error. The numerical analysis was performed in the Python programming language (Python Software Foundation, https://www.python.org).

#### Muscle activities

Due to the complex relationship between EMG signals and muscle forces and to the uncertainties in signals acquisition, the “on-off” timing of muscle activity is the simplest information obtainable from EMGs [34,35], which can be compared to muscle activations. For this reason, predicted muscle activities were qualitatively compared to the on-off timing of average EMGs from literature. In particular, the predicted activation levels of Gluteus Medius, Gastrocnemius Lateralis, Rectus Femoris, Biceps Femoris, and Tibialis Anterior were compared against the timing of EMGs of the same muscles reported for THR patients by Agostini et al. [36]. The comparison was carried out against the most common activation patterns reported for the patients 12 months after the surgery [36], in order to match the post-op conditions in which the motion-capture and HCF data described above were obtained.

### Muscle contribution study and geometry modifications

Muscle path uncertainties arising from estimated via-points, wrapping geometries, zero muscle thickness assumptions, and inaccurate local scaling can all be sources of errors in simulated HCFs. For this reason, the contributions of individual muscle elements to the three vector components of the HCF were analyzed to identify possible critical geometries.

**Fig 1:**
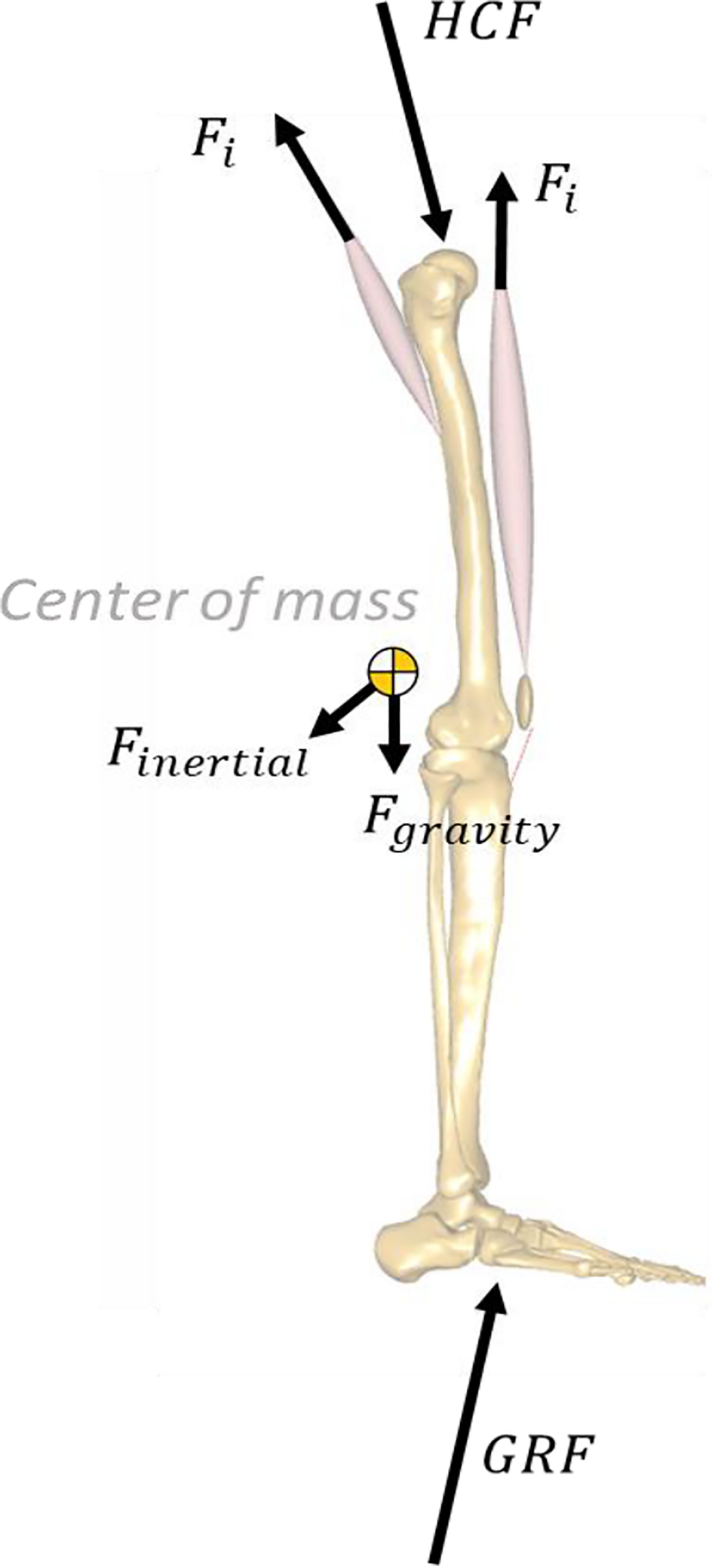
Free-body diagram of the leg for the calculation of muscle contribution to HCF. The directions of the force arrows are chosen to aid intuition. In reality, the sign convention for forces in Eq 1 should be fixed such that a positive force will always pull (or push) the system.

To calculate these contributions, the free-body diagram of a single limb from the hip down (Fig 1) was considered. Based on Newton’s first law, the following equation (Eq 1A) always held true—where *F*_*inertial*_ and *F*_*gravity*_ were the inertial (due to acceleration) and gravity forces on the limb, and *MF*_*hip*_ was the summation of the force vectors (*F*_*i*_) of the *M* individual muscle elements that crossed the hip joint (Eq 1B).

*F*_*inertial*_, *F*_*gravity*_ and GRF depended on model kinematics and mass distributions, and were not affected by muscle geometry or forces. Therefore *F*_*i*_ alone was an individual muscle’s contribution to the HCF.

$$HCF+F_{inertial}+F_{gravity}+GRF+{MF}_{hip}=0$$ (1A)

$${MF}_{hip}=\sum_{i=1}^{M}F_{i}$$ (1B)

Muscles in the model could assume piecewise linear or curved paths depending on the wrapping conditions. However, muscle paths were always linear in the sections where they crossed a joint that connected two segments, because a wrapping surface could only belong to one segment or the other. This permitted the definition of a muscle “line of action” as it crossed the hip joint—as it left the last point on the pelvis to connect to its first point on either the femur (mono-articular), tibia or patella (biarticular muscles).

From model kinematics, we calculated each muscle’s instantaneous line of action across the hip joint as a unit vector registered in the same reference frame as the measured hip forces. This vector was multiplied by the muscle’s force magnitude calculated by inverse dynamics to get the muscle’s HCF contribution—*F*_*i*_.

Muscles with: (a) high HCF contributions but relatively low joint moment contributions, or (b) a joint moment contribution higher than the overall joint net moment, were flagged as muscles that could potentially present errors in their geometrical definition. These flagged muscles were further investigated and compared to the MRI scans from the TLEM 2.0 cadaveric dataset [27] to verify the realism of their moment arms and lines of action. The comparison with the MRI scan was performed with the generic unscaled model, in order to align correctly the segmented muscle volumes with the model. If inconsistencies between muscle lines of action in the model and muscle volumes in the MRI scans were noted, the discretization of the muscle in the model were manually modified to better match the volumetric information. Additionally, all the remaining muscles spanning the hip joint were visually verified against the MRI data to identify any further incongruence that might have not appeared in the analysis of the contributions to the HCF.

#### Gluteus Maximus

**Fig 2:**
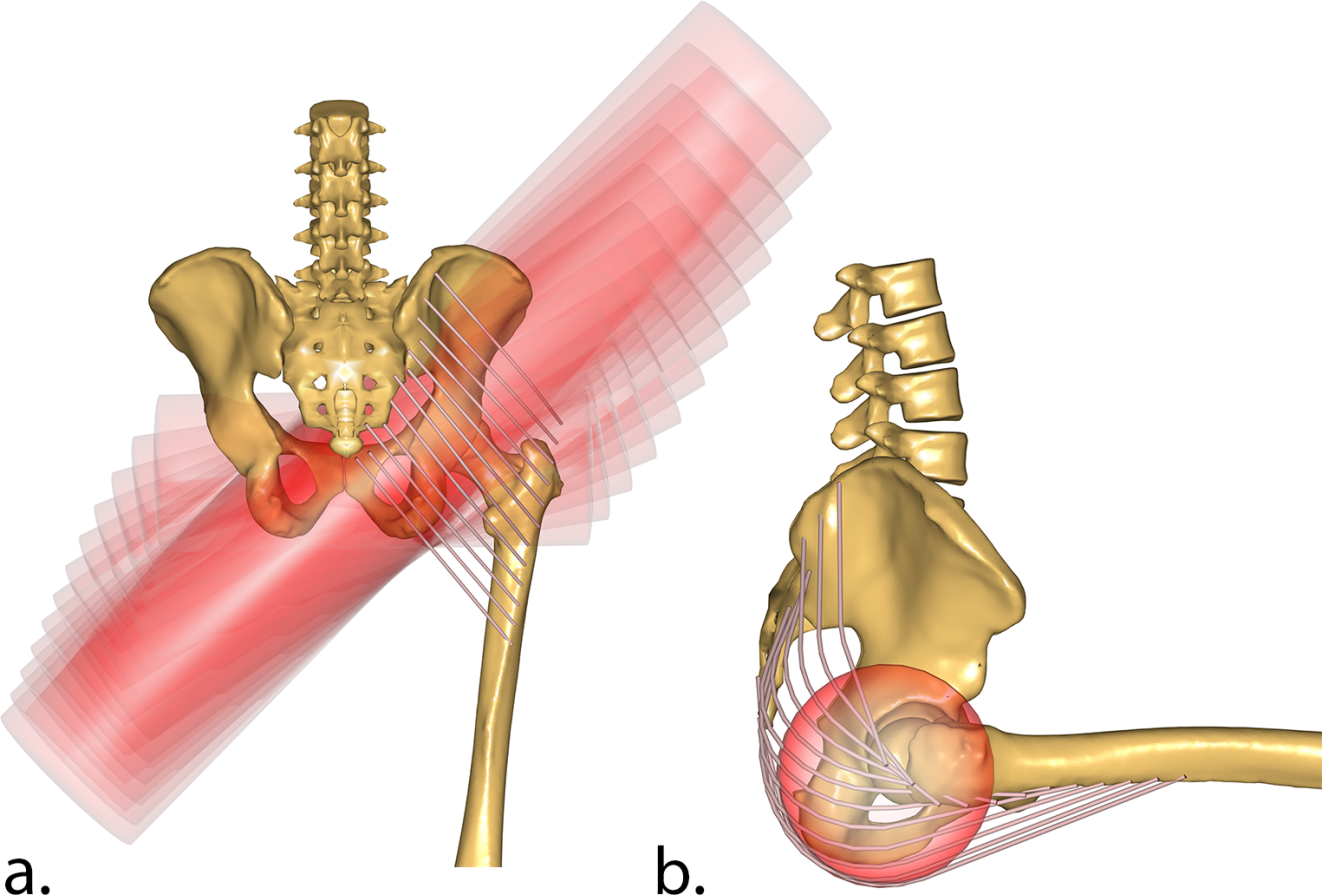
New wrapping surfaces for Gluteus Maximus. Each muscle element has its own wrapping cylinder (in red). The cylinders are aligned to match the overall muscle shape, and constructed to follow the underlying pelvis bone geometry. Figure *a*. (from posterior) show all the wrapping surfaces of the 12 strands of the muscle, while figure *b*. (sagittal) highlights a single cylinder for the most distal strand of the Gluteus Maximus Inferior.

Wrapping surfaces were introduced in the pelvic coordinate system to ensure that the Gluteus Maximus had anatomically realistic lever arms over the entire hip range of motion, with the same rationale described by Varady et al. [18]. The individual muscle elements were wrapped around cylinders fixed to the pelvic segment (Fig 2). However, instead of using a single cylinder as the original TLEM 2.0 implementation, we used individual cylinders for each muscle element. Each cylinder is oriented differently to capture the topology of the Gluteus Maximus anatomy in the original magnetic resonance imaging (MRI) scans. This reimplementation of the muscle wrapping with cylindrical surfaces has the same effect as wrapping the muscles over an ellipsoid, but with the added advantage that the muscle elements cannot slide off as the joint articulates, The use of independent cylindrical wrapping surfaces has also been suggested in literature by Rajagopal et al. [37], with the additional benefit of improving computational speed. Supporting information S9 Fig shows a comparison between old and new muscle elements.

#### Gluteus Medius and Minimus

**Fig 3:**
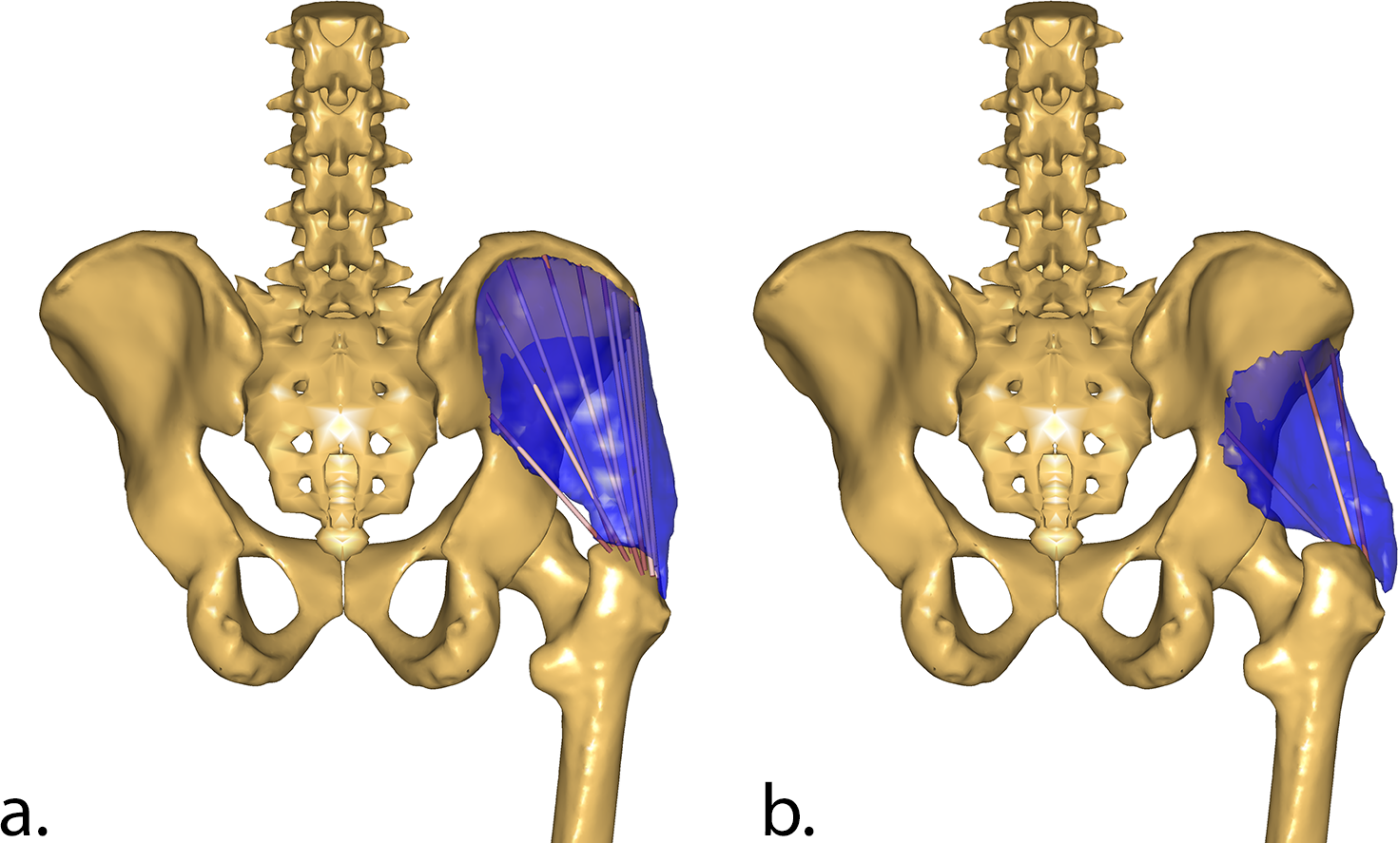
New origins for Gluteus Medius and Minimus. New origin points for the Gluteus Medius (a.) and Gluteus Minimus (b.) compared the segmented muscles (in blue) from the original cadaver MRI scans.

The topology of Gluteus Medius and Gluteus Minimus were modified by redistributing the elements composing these muscles. In particular, the origins of the muscle elements were redistributed to cover the entire span of the area of origin, moving the origins closer to the iliac crest to ensure the muscles had sufficient moment arms compared to the original cadaver MRI scans (see Fig 3 and S10 Fig).

#### Ilio-Psoas

**Fig 4:**
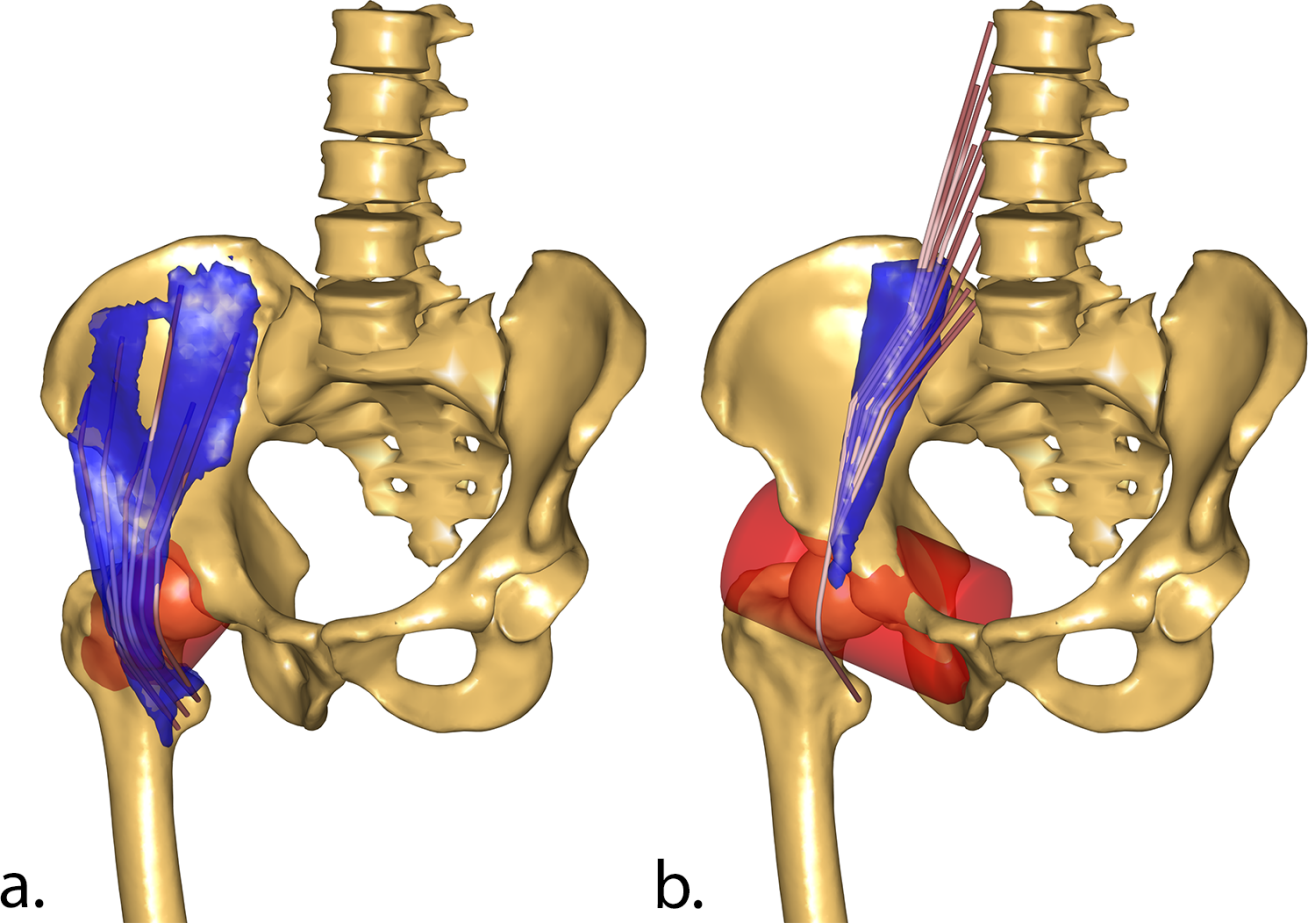
New wrapping surfaces for Iliacus and Psoas. New wrapping surfaces (red cylinder) for Iliacus (a.) and Psoas (b.) muscles compared to segmented muscles (in blue) from the original MRI scans.

With the original TLEM 2.0 implementation of the hip flexors, Psoas and Iliacus, the line of action at which they insert on the femur contributed to a high compressive force in the proximal/distal direction. In reality, these muscles wrap over the anterior inferior iliac spine on the pelvis. In the TLEM 2.0 model, the whole Ilio-Psoas muscle group was only represented by a single point where the muscles left the pelvis segment. In the new implementation, cylindrical wrapping surfaces was introduced (Fig 4), changing the line of insertion and the component of the muscle force that contributed to the proximal/distal compressive force while generating only a small flexion moment. The Iliacus wrapping cylinder was fixed to the femur coordinate system, while the Psoas wrapping cylinder was added in the pelvis coordinate system.

#### Knee flexors

**Fig 5:**
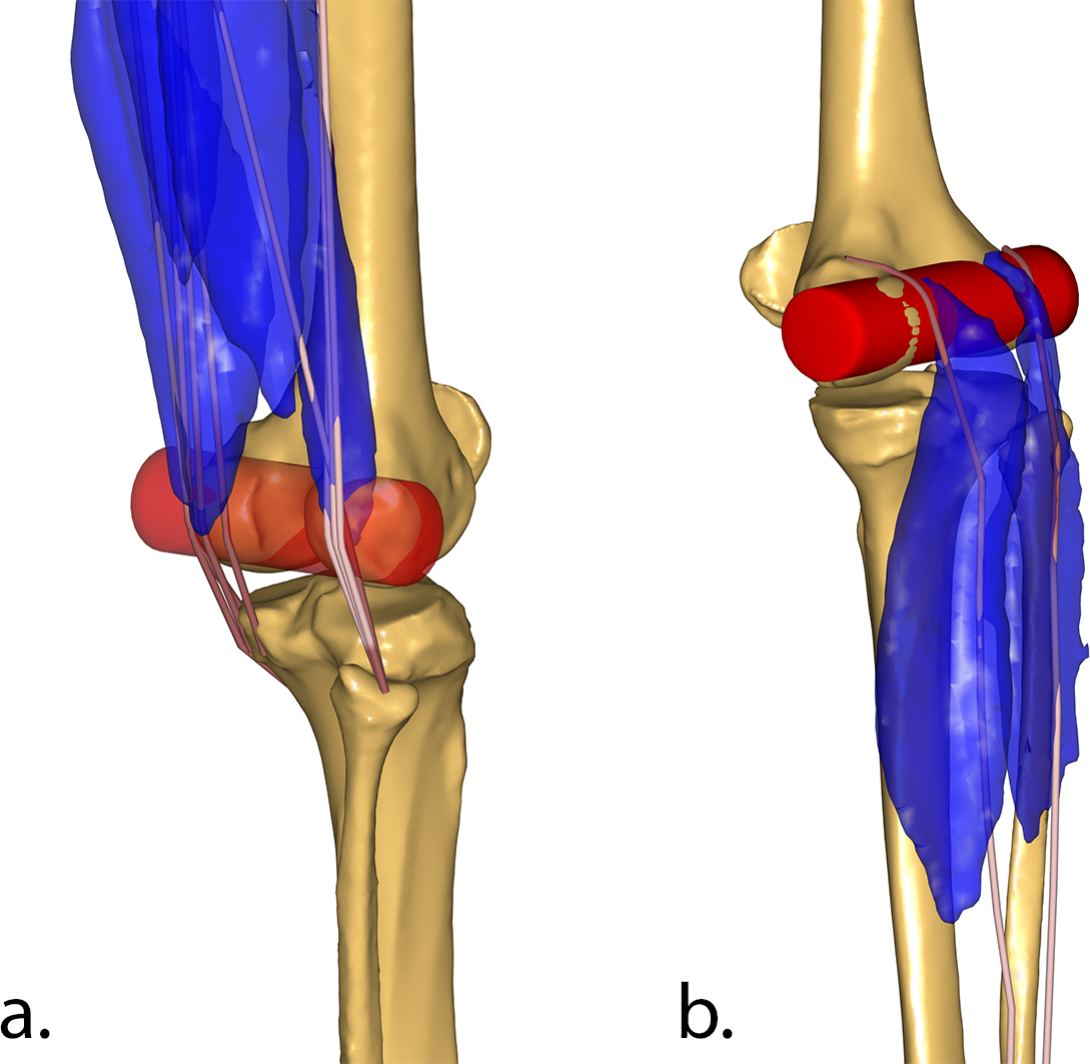
New wrapping surfaces for knee flexors. Wrapping cylinder (in red) of Semimembranosus, Semitendinosus, and Bicep Femoris elements (a.), and Gastrocnemius (b.) around the femoral condyles, compared to the segmented muscles from the original cadaver MRI scans (in blue).

The lines of action of the Semitendinosus, Semimembranosus, Biceps Femoris, and Gastrocnemius were modified, and two wrapping cylinders were introduced around the posterior femoral condyles to match the insertion areas of the muscles around the knee (Fig 5). This represents an important change, since the knee flexors in the original TLEM 2.0 dataset had almost no moment arm when the knee was fully extended. All new wrapping cylinders were fixed to the femur coordinate system.

#### Tensor Fasciae Latae

**Fig 6:**
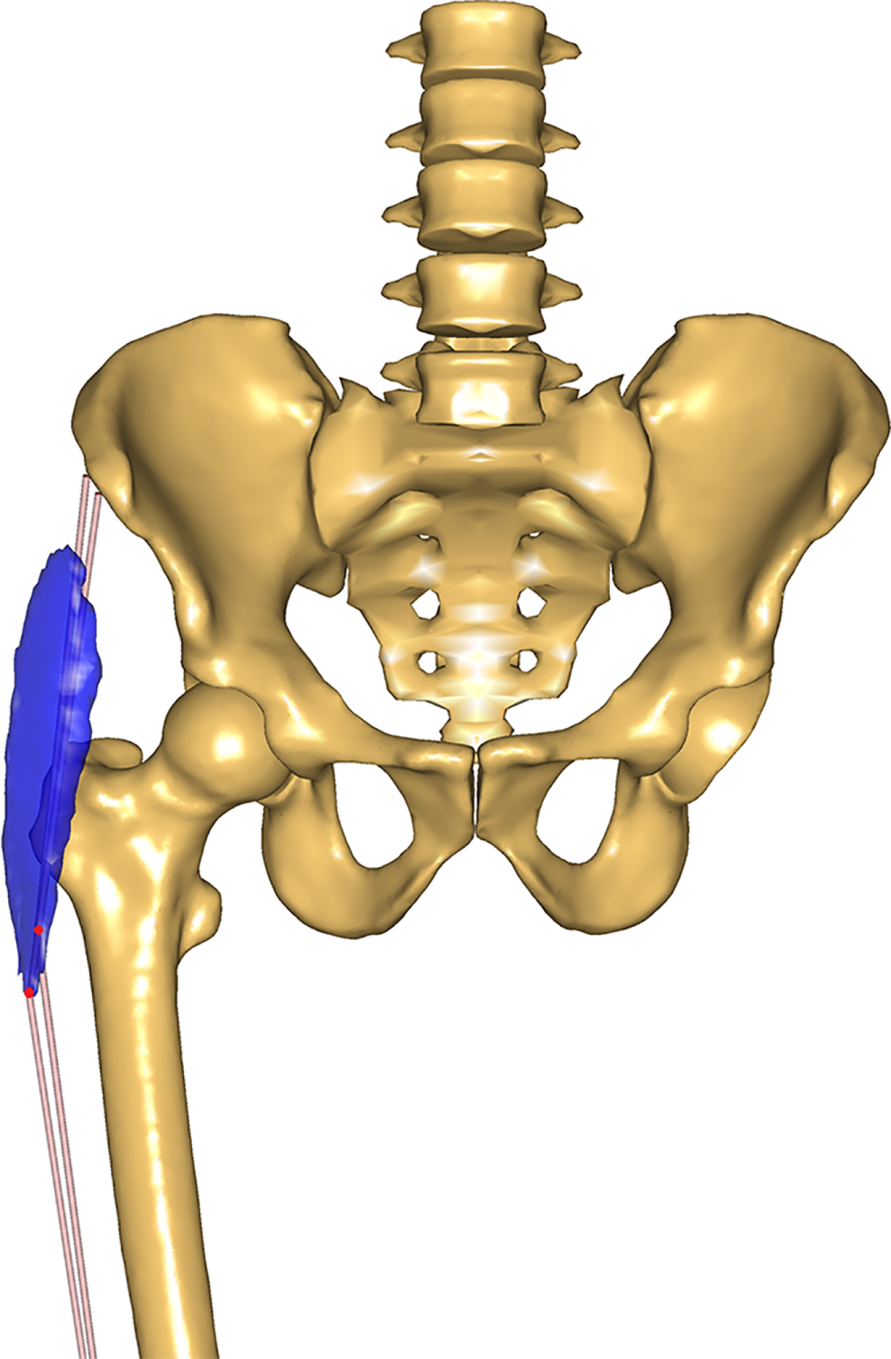
New line of action for Tensor Fasciae Latae. New lines of action for Tensor Fasciae Latae elements with muscle path points defined inferiorly to the surface of the trochanter.

The muscle paths were changed by adding muscle path points (via points) inferiorly to the surface of the trochanter to imitate the insertion of the Tensor Fasciae Latae on the Iliotibial band (Fig 6).

## Results

### Muscle contribution study

The Bicep Femoris initially showed a contribution to hip extension during the swing phase higher than the overall net internal extension moment, indicating that a substantial amount of co-contractive activity of the hip flexors would have been necessary to match the total net moment, therefore increasing the overall contact forces. This prompted the investigation of the wrapping of the hamstrings around the condyles of the femur, where the introduction of a more accurate wrapping surface led to a reduction in HCFs of up to 0.40*BW during the swing phase.

The wrapping surfaces introduced for Iliacus and Psoas enabled a slightly higher contribution of these two muscles to the hip flexion moment, which also led to a small increase in their contribution to their contact forces. Presumably, this was because a better lever arm made them preferentially activated, thus increasing their contribution.

The wrapping surfaces introduced for the Gluteus Maximus did not alter their contribution to the total contact force and only slightly reduced the extension moment contribution during stance. The contribution of the superior section to the abduction moment was also increased, while the inferior compartment slightly adducts the joint in the very early stance phase. The Gluteus Medius contribution to the HCF was reduced over the entire stance phase as a consequence of a lower contribution to the hip abduction moment. This was partially compensated by a higher activation of the Gluteus Minimus, which showed higher contribution to both contact forces and abduction moment.

Graphs of the muscle contribution to the hip moments and joint contact forces are available as Supporting Information (S1–S8 Figs).

### Hip contact forces

**Fig 7:**
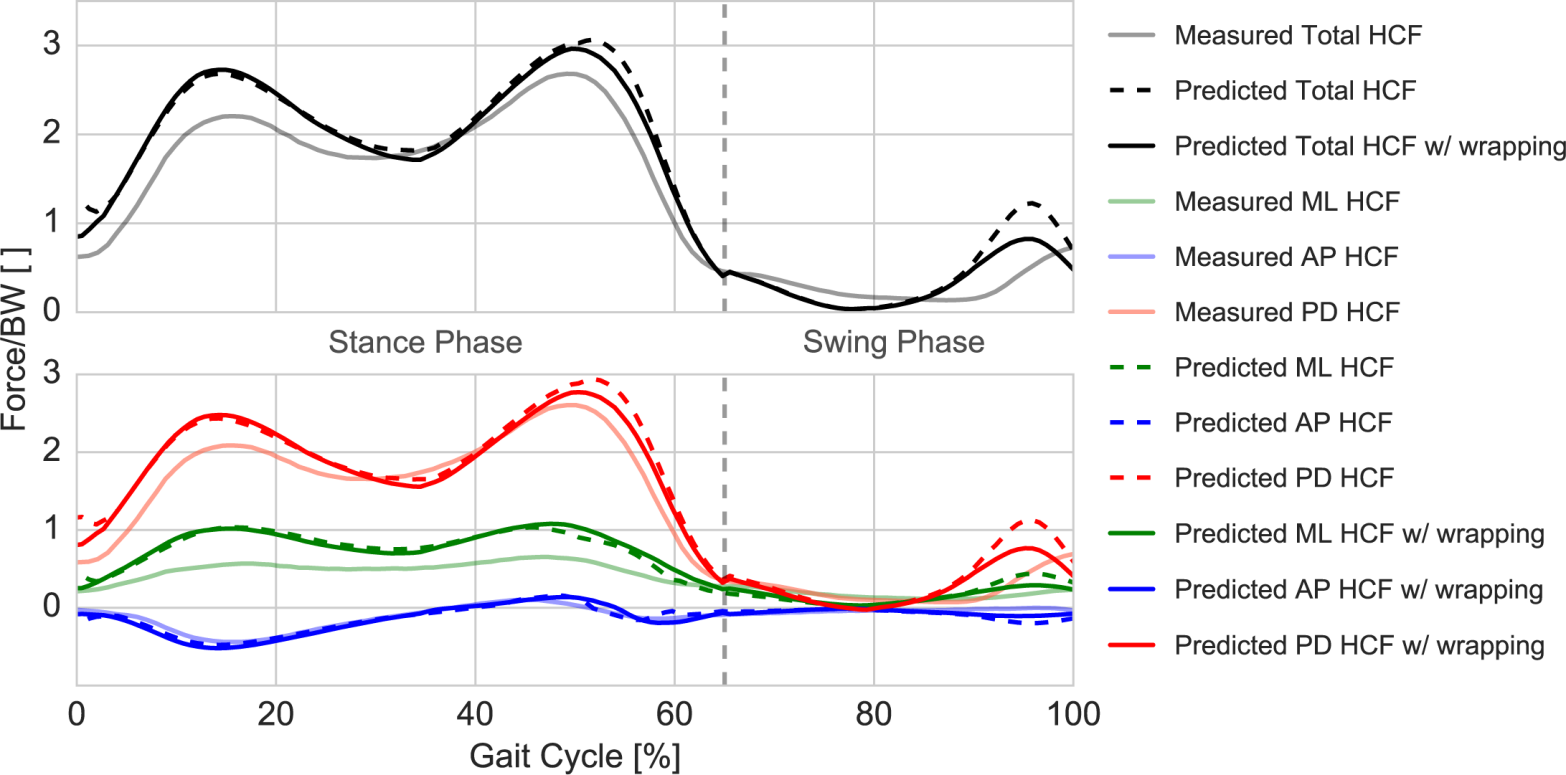
Predicted and measured HCF over a gait cycle. HCF predicted by the musculoskeletal model before (dashed lines) and after (solid dark lines) muscle wrapping modifications compared to the measured HCF from the instrumented implant (solid shaded lines) [8,29]. The upper plot reports the total HCF magnitude (in black), while the bottom one reports the single HCF components: medio-lateral (MD) in green, antero-posterior (AP) in blue, and proximo-distal (PD) in red.

The predicted HCF showed good agreement with the overall trend and timing of the measured HCF from the instrumented prosthesis (Fig 7). The RMSE, calculated for the total HCF and evaluated over the whole gait cycle, was found to be 0.298*BW, while the RMSE evaluated separately for stance and swing phase was 0.337*BW and 0.211*BW respectively.

The post-refinement error was, however, non-uniformly distributed over the three directional components. The analysis of the individual force components showed good agreement for the antero-posterior component with an RMSE of 0.054*BW, while the proximo-distal (RMSE = 0.243*BW) and medio-lateral (RMSE = 0.267*BW) components were over-predicted mainly during the stance phase.

As a comparison, the pre-refinement RMSE value was 0.359*BW over the whole gait cycle (0.372*BW and 0.342*BW for stance and swing phase, respectively). The RMSE for the single force components were 0.088*BW, 0.312*BW and 0.267*BW in the antero-posterior, proximo-distal and medio-lateral directions, respectively.

The maximum error during the stance phase was associated with its first peak (loading phase), where the resultant force (2.72*BW) was over-predicted by 0.50*BW (vs. measured peak force 2.20*BW) and remained comparable to the pre-refinement peak prediction (2.69*BW), while the error associated with the second peak (terminal phase) was 0.28*BW (2.96*BW vs 2.68*BW), showing some improvement from the pre-refinement peak value (3.06*BW).

### Muscle activity

**Fig 8:**
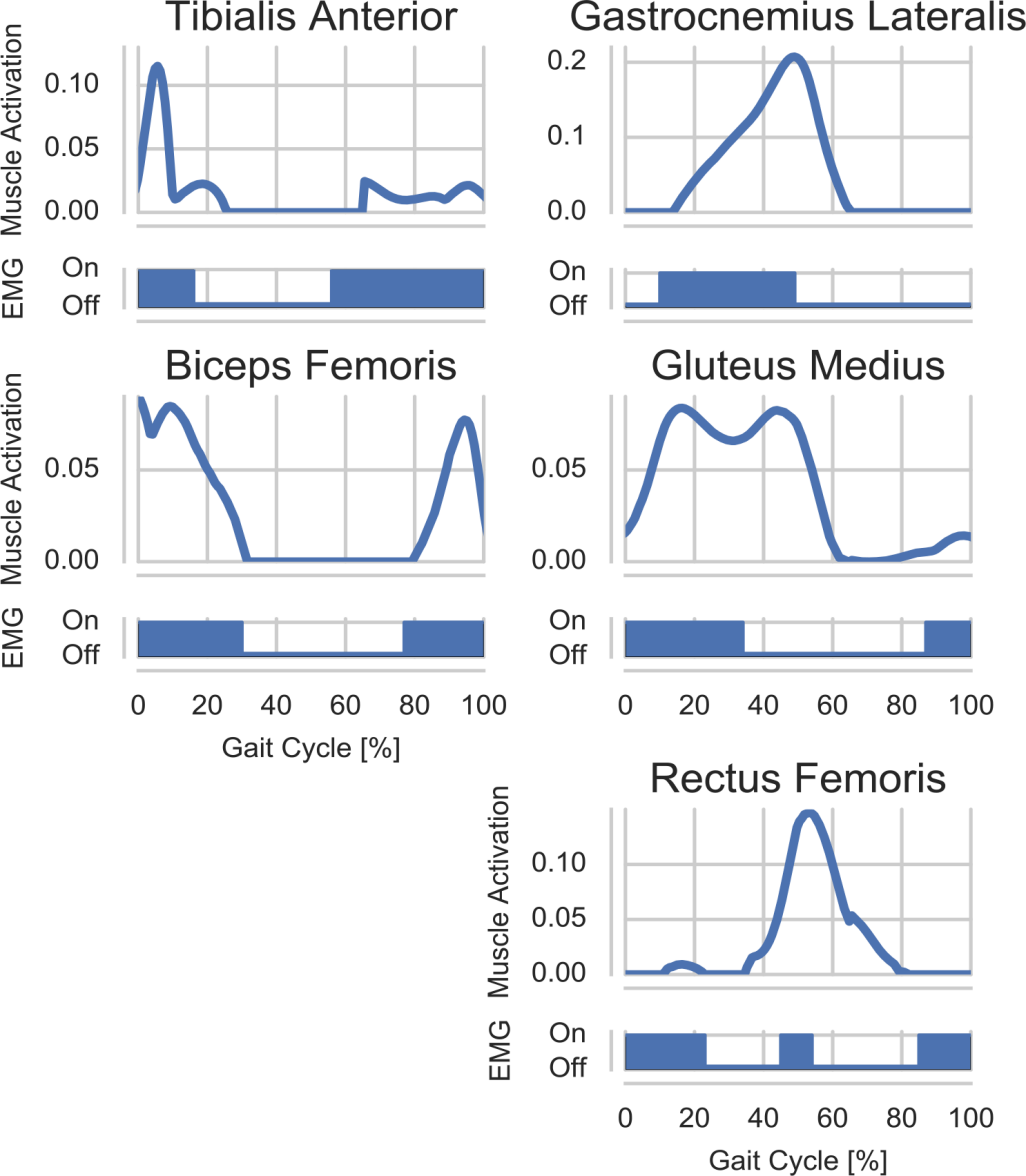
Predicted muscle activations during gait vs. average timing of EMGs. Comparison between predicted muscle activations and average “on-off” timing of EMG signals reported for THR patients by Agostini et al. [36].

The Tibialis Anterior was active in the simulations during the swing phase until the loading phase of the stance, with similar duration, onset and offset timing compared to the average EMG signal (Fig 8). A similar activation pattern was also predicted for the Biceps Femoris, which was active from mid-swing phase to mid-stance phase, in agreement with the corresponding EMG. The Gastrocnemius was active from the loading phase of the stance until toe-off, with a slightly longer and delayed activity compared to the EMG. The predictions for the Gluteus Medius showed activity from the end of the swing phase throughout the stance phase, with a similar onset compared to the EMG but with a delayed offset. The Rectus Femoris on the other hand was briefly active during the loading response of the stance and again from late stance phase to mid-swing phase, whereas the average EMG used as a comparison showed activity from mid-swing phase to mid-stance phase and only a short activation in the late stance phase. Compared to the activation timing before the introduction of the new wrapping, only the Biceps Femoris showed some differences, with the new configuration better matching the EMG timing during the mid-stance phase. The remaining muscles did not show substantially altered recruitment patterns in terms of timing. For further consideration, refer to the muscle force contributions figures provided as Supporting Information (S1–S7 Figs), in which the timing of the reported muscle forces corresponds to the activation timing of the muscles.

## Discussion

This study documented the development of a lower limb musculoskeletal model, with a particular focus on the refinement of the geometry of the muscles spanning the hip. The definition of muscle wrapping was identified as a major factor determining the accuracy of HCF prediction.

The quantitative validation of the model provides an estimation of the error associated with HCF prediction. The results of a single gait cycle showed overall a good agreement between predicted and measured HCF, with an average RMSE of 29.8%BW, which lies within the lower end of RMSE range (23.2–66.3%BW) reported in the literature for similar models [23,38–40]. In particular, the results of our refined model compare well with the previous TLEM 1.1 model, which presented a RMSE of 40–43%BW with the same muscle modelling set-up [40]. Nevertheless, a certain amount of over-prediction persists during the loading response of the stance phase, particularly in proximo-distal and medio-lateral directions.

The detailed study of individual muscle contributions to the HCF succeeded in identifying muscles with incorrect anatomy, which would have been difficult to intuitively identify otherwise. For example, excessive HCF contributions of the bi-articular hamstrings were due to insufficient moment arms at the knee joint, instead of the hip, which resulted in high forces during knee deceleration in the swing phase. Refining the geometries of the muscles thus identified reduced RMS errors on HCF magnitudes by 17% (from 0.359*BW to 0.298*BW) over the whole gait cycle, with a maximum reduction in the HCF error of 56% (from an error of 0.710*BW to 0.309*BW, at 78% of the gait cycle).

It is important to stress that the contribution of the different muscles to the overall HCF was only used as a flag to identify potential errors in the geometrical definition of the muscles. The model was not tuned to a desired response. The refinement of the muscle lines of action was carried out in order to better resemble the volumetric information from the cadaveric MRI scans of the TLEM 2.0 dataset, and not to match the HCF data from the instrumented implant. This is particularly relevant in the development of a generic model, as the risk of overfitting the model for this specific application could lead to worse outcomes in different applications. The modifications implemented on the muscle elements were based on MRI scans obtained for a single pose of the cadaver and, therefore, cannot guarantee accurate matching throughout the overall range of motion of the hip joint. However, further analysis of the muscle lever arms, computed over an extended range of hip flexion, abduction, and external rotation angles, revealed trends similar to those reported in literature [41] (S11–S15 Figs). The current model implementation seems to provide the modified muscles with sufficient lever arm for a range of hip angles that spans from 20°extension to 90° flexion, from 30° adduction to 40°abduction, and from 40° internal rotation to 40° external rotation. Within this range, the muscles do not present any evident discontinuity in their hip lever arms, rather than engaging or disengaging from their wrapping surfaces. Only the elements of Gluteus Maximus Superior present a sudden drop in their abduction moment arm for abduction angles greater than 40°, which represents a limitation in the range of validity of this model. Nevertheless, there seem to be no clear indication that would prevent the use of this model for more complex activities within the aforementioned range of motion. However, further validation of the model is necessary before its application for activities other than gait. The results also indicate that a reduction in proximo-distal and medio-lateral HCF validation errors should eventually involve more substantial changes in the contribution of Gluteus Medius, which was shown to be the primary, dominant contributor to hip contact forces during gait (S8 Fig). The exact nature of this change, however, remains unclear at this stage. Although the current muscle paths appear to comply well with the MRI muscle scans (Fig 3), its lever arm could have also been affected by an incorrectly defined hip joint centre location.

Post-operative muscle activations for THR patients have been previously reported to differ from those acquired from healthy subjects [36,42,43], therefore in this study, the predicted activities were compared to reported EMG signals from a similar cohort of subjects. The comparison between predicted muscle activations and the timing of the EMG signals showed an overall good agreement for the investigated muscles. The activities of Tibialis Anterior, Gastrocnemius Lateralis and Biceps Femoris had similar activation patterns compared to the reported EMG signals. The predicted activity for the Gluteus Medius had a comparable onset time to the average EMGs, but it continued throughout the stance phase. This activation pattern is in agreement with previously reported EMG patterns for the Gluteus Medius in the literature [44]. The Rectus Femoris activity, on the other hand, did not perfectly match the on-off pattern of the reported EMGs. The predicted activation, however, are in agreement with fine-wire EMG measurements [45] and the predictions of previous musculoskeletal models [23,26,38,46]. Despite the known limitations of comparisons with EMG signals [13], the model presented in this work seem to be in agreement with the literature and to predict realistic muscle activations during gait. Although conclusions could not be gathered regarding the magnitudes of muscle activities and forces, the good agreement with the EMG timing and between measured and predicted HCF points toward an overall realism of the predicted muscle forces, whose knowledge could have important clinical applications.

The major limitation of this study is the validation against a single gait trial from a single patient, due to the limited public availability of complete input data. The release of additional data samples from different patients would serve as a benchmark to test the model with different kinematic and anthropometric inputs. Fischer et al. [40] had access to a set of ten gait trials from different patients with instrumented prostheses, allowing them to validate the previous TLEM 1.1 model and prove his robustness against a larger anatomical variability in the patients. A similar evaluation must be performed also for the improved model (based on TLEM 2) proposed in this paper.

Moreover, no information on the subject-specific bone geometry was available and therefore not taken into account in the modelling workflow. This information could have reduced the potential sources of uncertainty and further improved the model’s predictions. In particular, information regarding the pelvis dimensions and radiographic images of the markers’ position relative to the bony landmarks would have allowed for a more accurate scaling of the pelvis and determination of the hip joint center position [47–49], which could have affected the computation of joint moments, muscle lever arms, and therefore muscle and joint forces.

Regarding the development of a generic lower-limb model, one limitation is the fact that the TLEM 2.0 model was built on the MRI images of a single cadaver and therefore cannot take into account the variance in muscular geometry present in the overall population. As the focus of this study was placed on the muscles of the thigh, with hip contact forces as a validation measure, the knee was modelled as a simple 1-DOF hinge joint. This simplification could potentially introduce kinematic and kinetic errors, which would affect the behavior of the thigh muscles, and therefore the forces computed at the hip. Nevertheless, this simplification has been successfully adopted in the past [21] and it should not pose a serious cause for concern. Similarly, the muscles in the model were characterized by an isometric strength independent of their length. Although this did not have any pronounced influence on the hip contact force in our case with a normal gait trial, it may not hold true when modelling more complex activities.

The tools and working logic that were developed in this framework allow for separate evaluation of different potential sources of error. While certain muscle geometries might need further refinement, future efforts will also focus on investigating other known potential sources of error [50], including muscle coordination strategies [51], uncertainty in hip location [52,53] and kinematics [54] associated with markers’ positions and soft tissue artefacts [55]. Finally, it must be kept in mind that the validation of musculoskeletal models is a continuous process, and therefore also this model should be specifically validated for any new application [20]; in its current form, it cannot yet be considered validated against the variability in anatomy and kinematics that can be encountered when modelling different activities or different patients, and future users are therefore advised to always verify the validity of its predictions.

## Conclusion

To provide accurate predictions, musculoskeletal models require a high level of detail for bone and muscle geometry, joint position and markers’ kinematics. This study focused on refining a generic lower-limb muscle geometry verified with MRI scans that would minimize errors associated with incorrect modelling of muscle paths and lever arms, particularly of glutei, ilio-psoas, tensor fasciae latae, and knee flexors. Although most of the improvement was observed during the swing phase and a certain over-prediction persists during the stance phase, a satisfactory level of geometrical accuracy of muscle paths has been achieved with the refinement of this model. An important source of inaccuracy and error has therefore been identified and a detailed strategy outlined to improve prediction accuracy.

## Supporting information

S1 Fig Hamstring contribution to hip internal flexion moment. (TIF)

S2 Fig Hamstring contribution to total hip contact force. (TIF)

S3 Fig Ilio-Psoas contribution to hip internal flexion moment. (TIF)

S4 Fig Ilio-Psoas contribution to total hip contact force. (TIF)

S5 Fig Glutei contribution to hip internal flexion moment. (TIF)

S6 Fig Glutei contribution to hip internal abduction moment. (TIF)

S7 Fig Glutei contribution to total hip contact force. (TIF)

S8 Fig Contribution of newly modified muscles to total hip contact force. (TIF)

S9 Fig Gluteus Maximus elements before and after refinement at different angles of hip flexion. The Gluteus Maximus elements in the model are visualized from a posterior and a lateral view at different angles of hip flexion: 0°, 10°, 40°, and 90°.
(TIF)

S10 Fig Gluteus Medius elements before and after refinement. Gluteus Medius elements in the model are visualized in pink and overlapped with segmented muscle volumes (in blue) and contours of the origin and insertion areas (red dots) from the MRI scans.
(TIF)

S11 Fig Gluteus Maximus elements moment arms during hip flexion, abduction and external rotation. Moment arms are reported for the different elements of Gluteus Maximus over a range of hip flexion (+)/extension (-), abduction (+)/ adduction (-), and external (+)/ internal (-) rotation angles. In brackets the number of elements constituting the muscle is reported.
(TIF)

S12 Fig Gluteus Medius and Minimus elements moment arms during hip flexion, abduction and external rotation. Moment arms are reported for the different elements of Gluteus Medius and Minimus over a range of hip flexion (+)/extension (-), abduction (+)/ adduction (-), and external (+)/ internal (-) rotation angles. In brackets the number of elements constituting the muscle is reported.
(TIF)

S13 Fig Iliopsoas elements moment arms during hip flexion, abduction and external rotation. Moment arms are reported for the different elements of Iliacus and Psoas over a range of hip flexion (+)/extension (-), abduction (+)/ adduction (-), and external (+)/ internal (-) rotation angles. In brackets the number of elements constituting the muscle is reported.
(TIF)

S14 Fig Hamstrings elements moment arms during hip flexion, abduction and external rotation. Moment arms are reported for the different elements of Semimembranosus, Semitendinosus, and Biceps Femoris over a range of hip flexion (+)/extension (-), abduction (+)/ adduction (-), and external (+)/ internal (-) rotation angles. In brackets the number of elements constituting the muscle is reported.
(TIF)

S15 Fig Tensor Fasciae Latae elements moment arms during hip flexion, abduction and external rotation. Moment arms are reported for the different elements of Tensor Fasciae Latae over a range of hip flexion (+)/extension (-), abduction (+)/ adduction (-), and external (+)/ internal (-) rotation angles. In brackets the number of elements constituting the muscle is reported.
(TIF)
