# Supplementary figures and images for: Refining muscle geometry and wrapping in the TLEM 2 model for improved hip contact force prediction
Source: PLoS One. 2018 Sep 17;13(9):e0204109. doi: 10.1371/journal.pone.0204109 (PMC6141086; doi:10.1371/journal.pone.0204109)

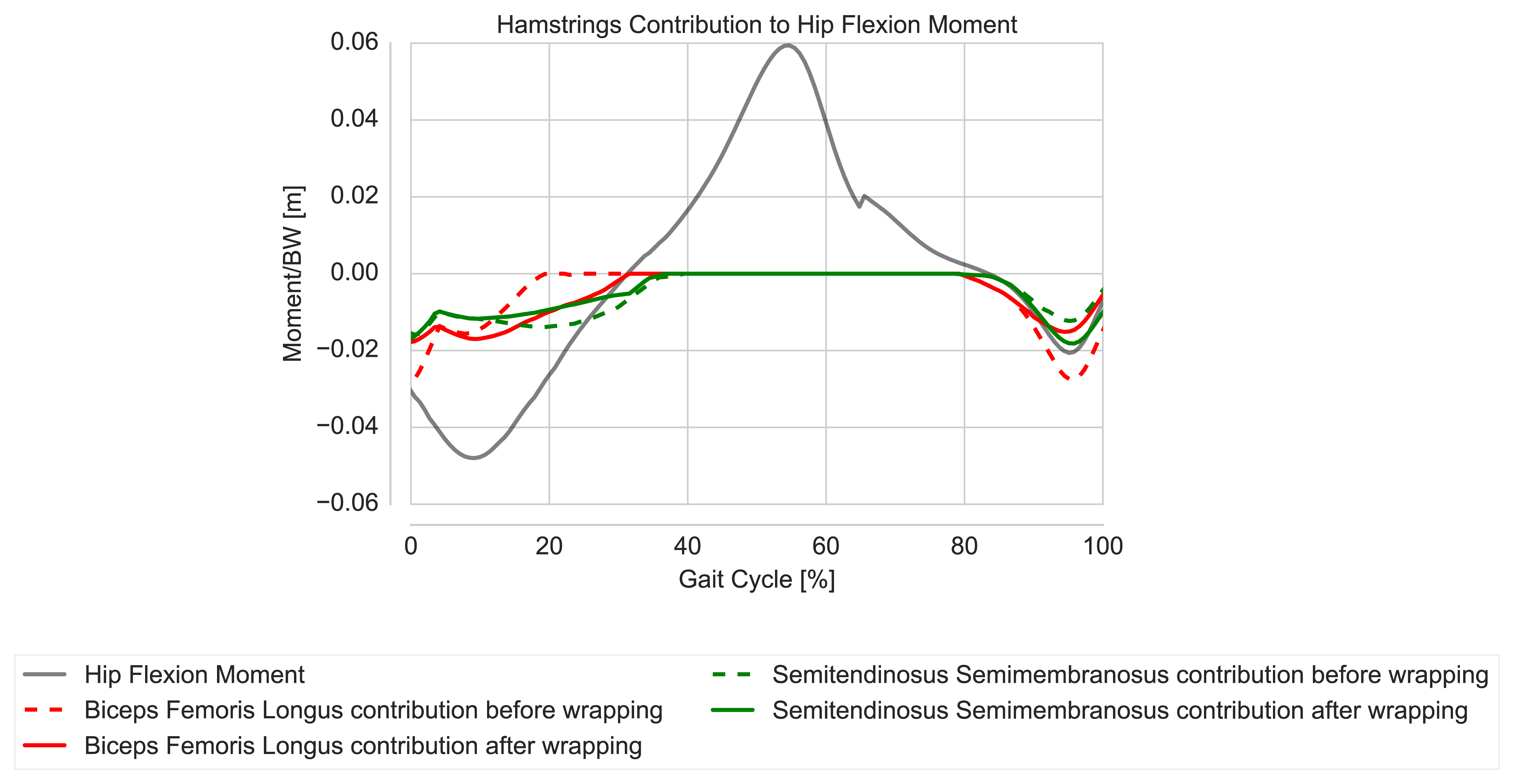

Supplement: S1 Fig — (TIF) [file pone.0204109.s001.tif]

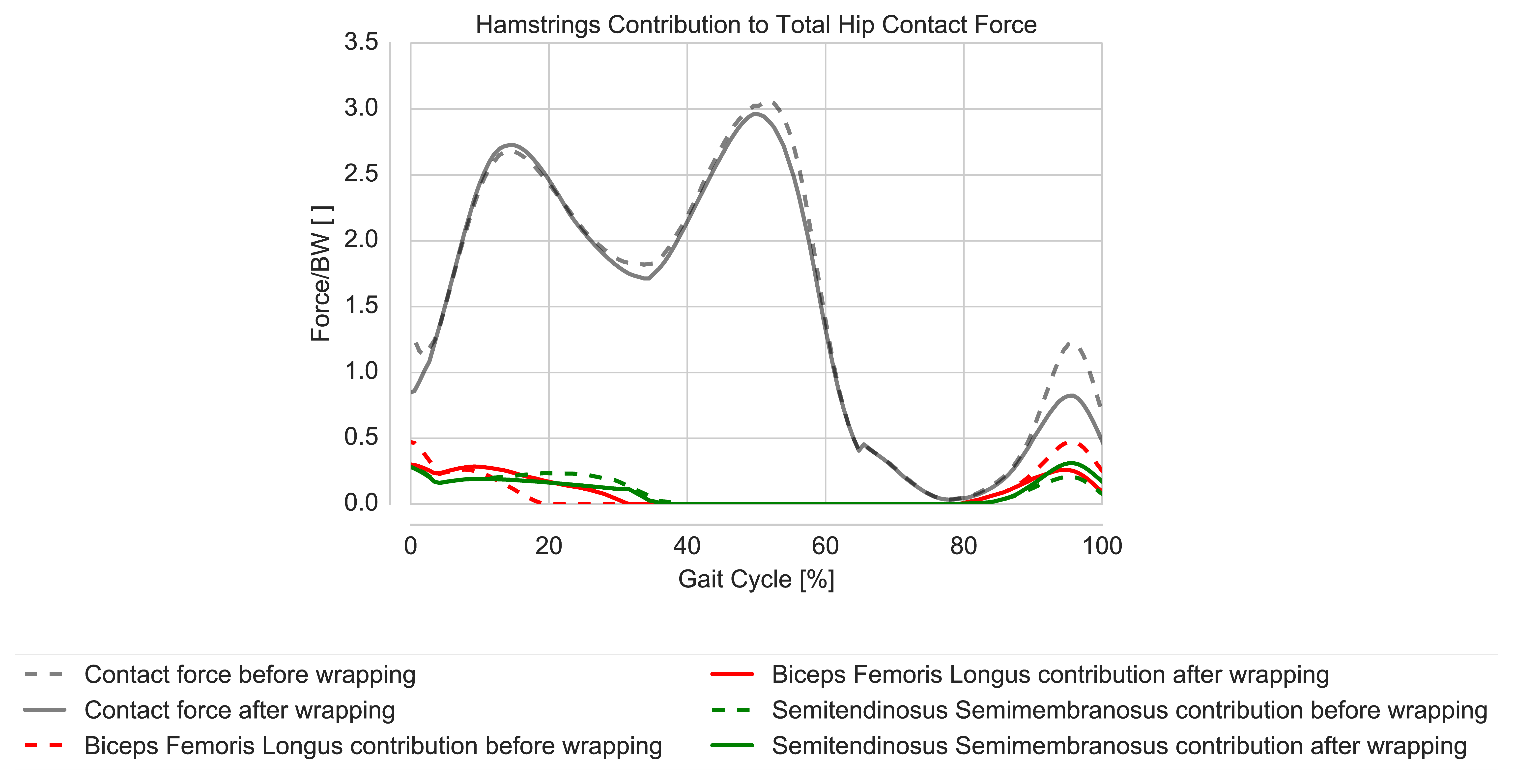

Supplement: S2 Fig — (TIF) [file pone.0204109.s002.tif]

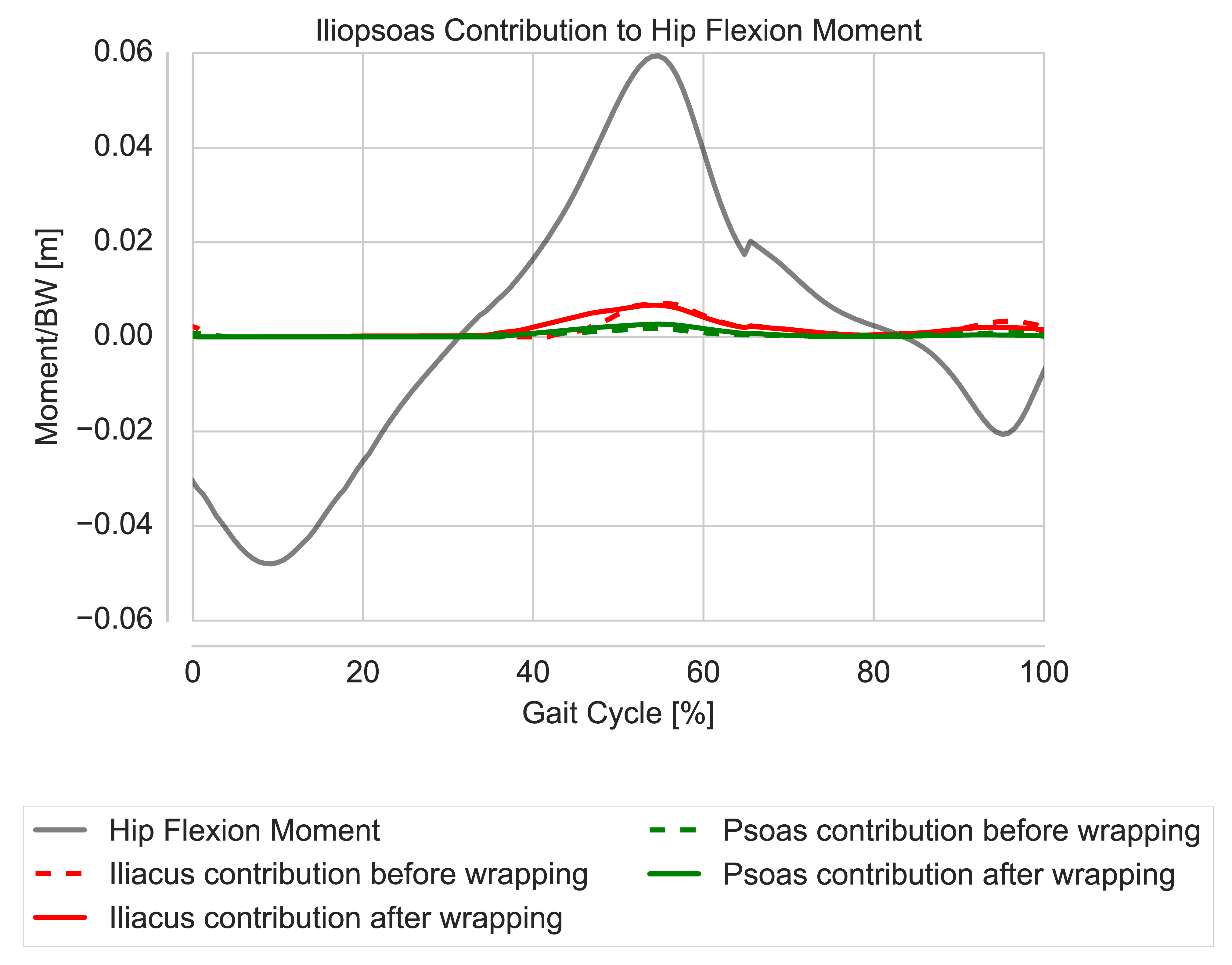

Supplement: S3 Fig — (TIF) [file pone.0204109.s003.tif]

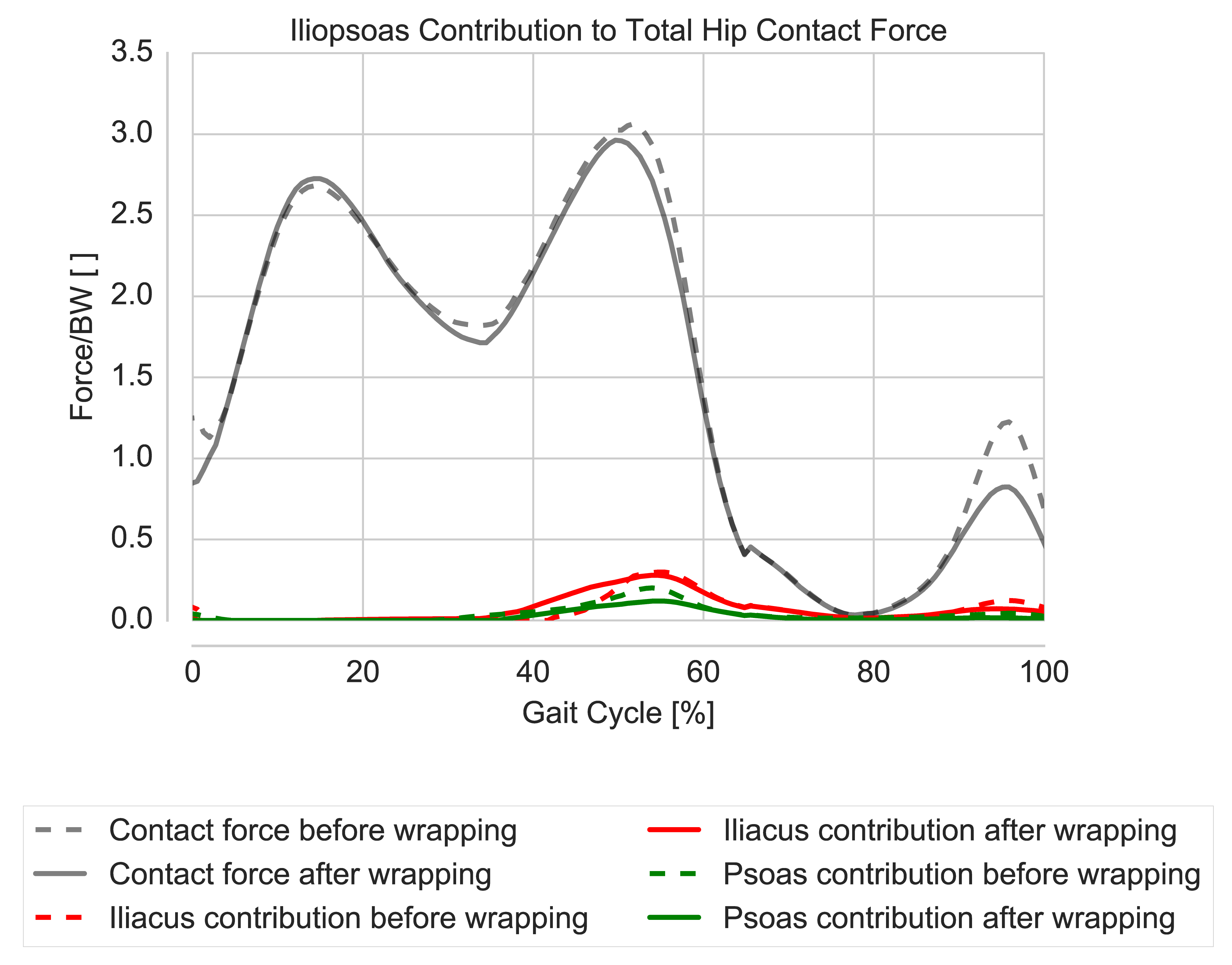

Supplement: S4 Fig — (TIF) [file pone.0204109.s004.tif]

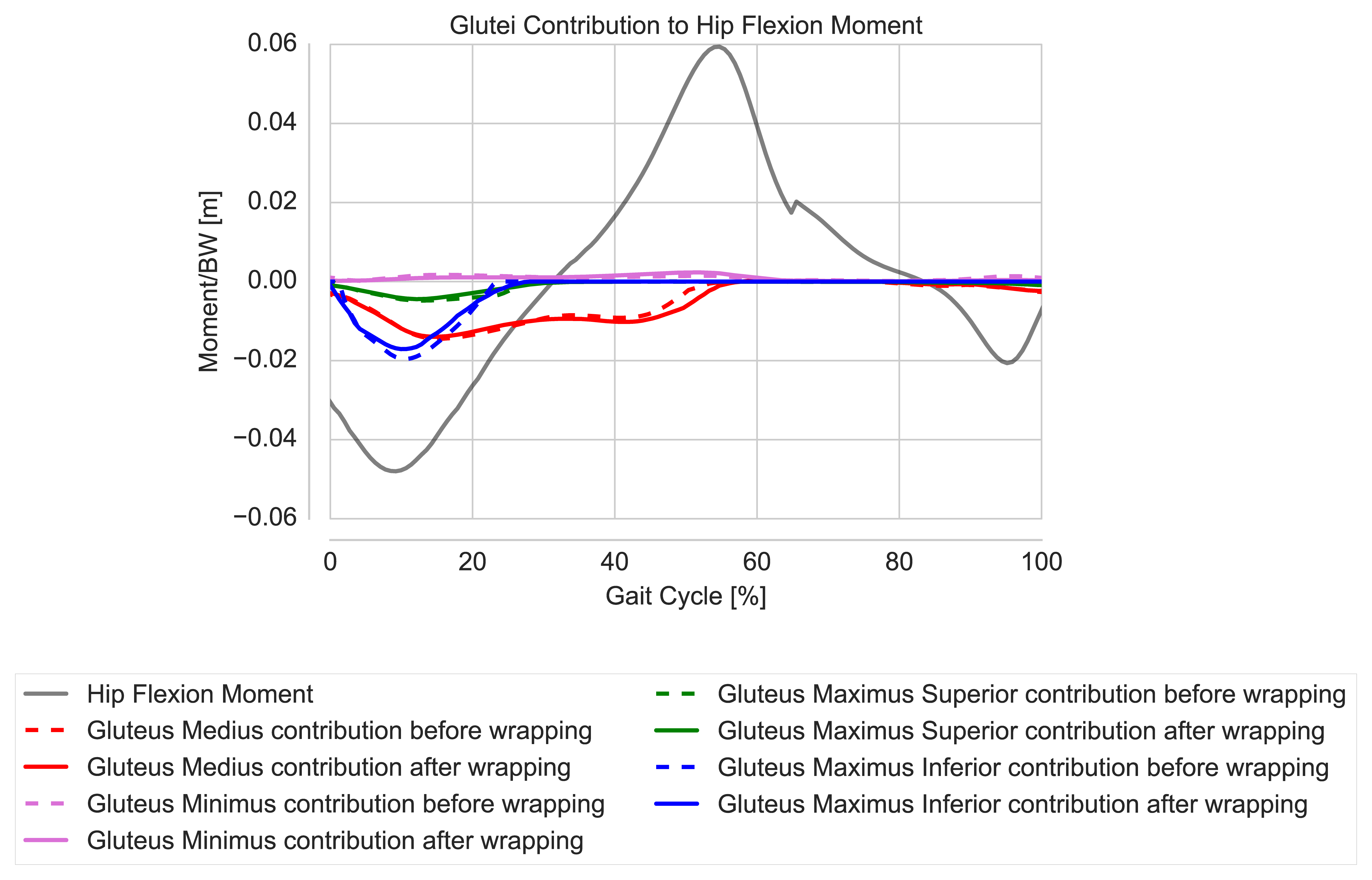

Supplement: S5 Fig — (TIF) [file pone.0204109.s005.tif]

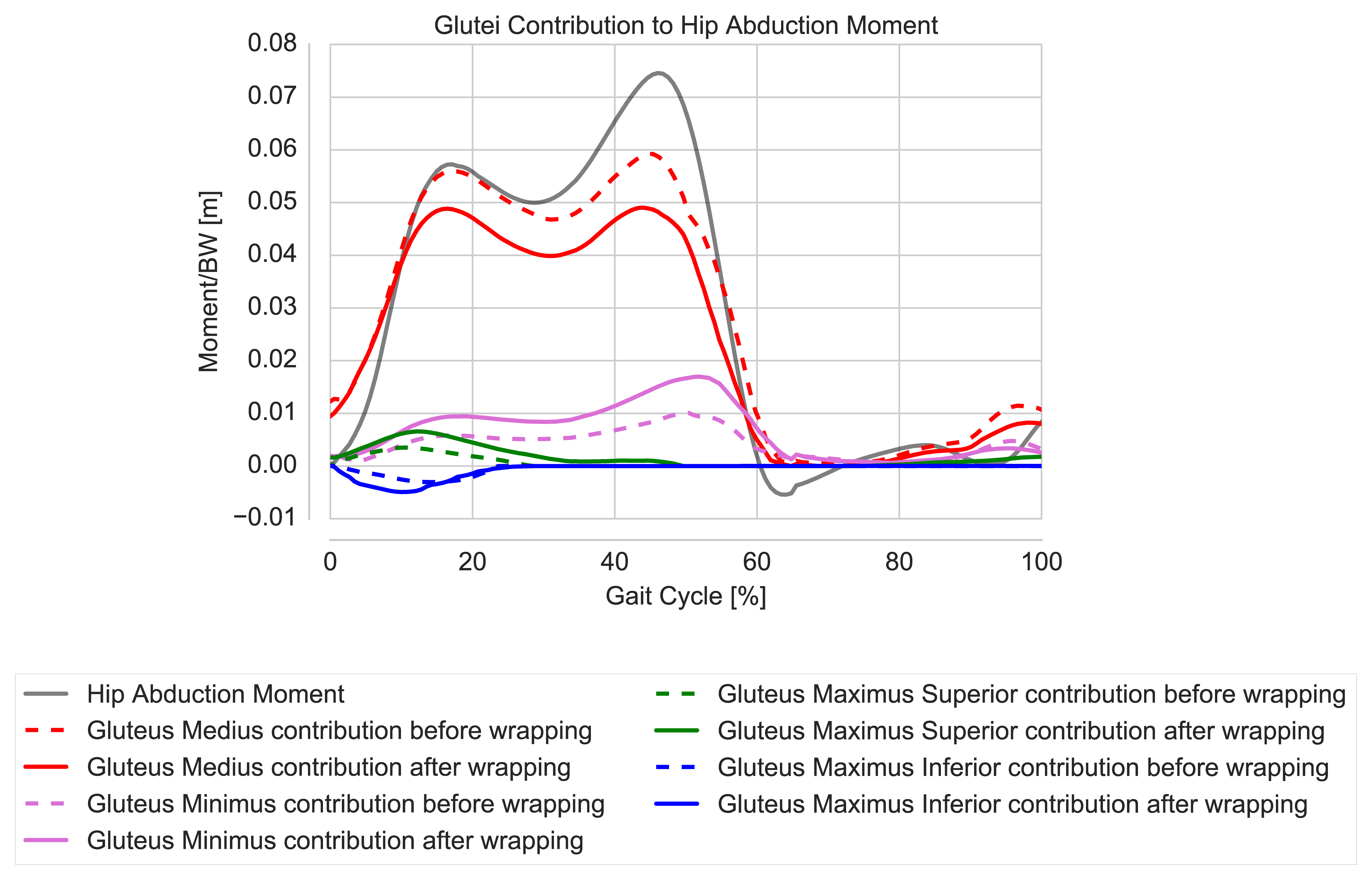

Supplement: S6 Fig — (TIF) [file pone.0204109.s006.tif]

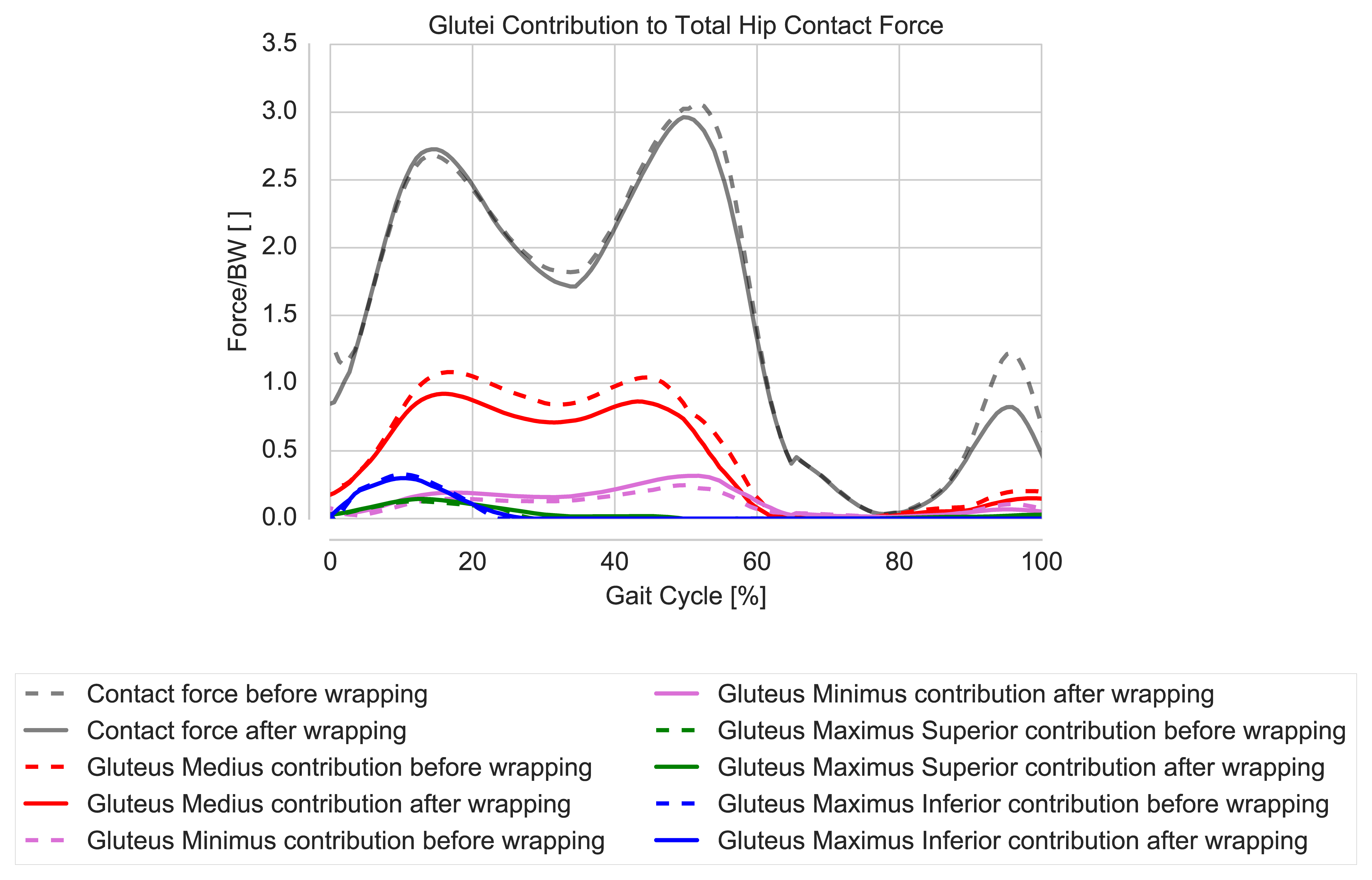

Supplement: S7 Fig — (TIF) [file pone.0204109.s007.tif]

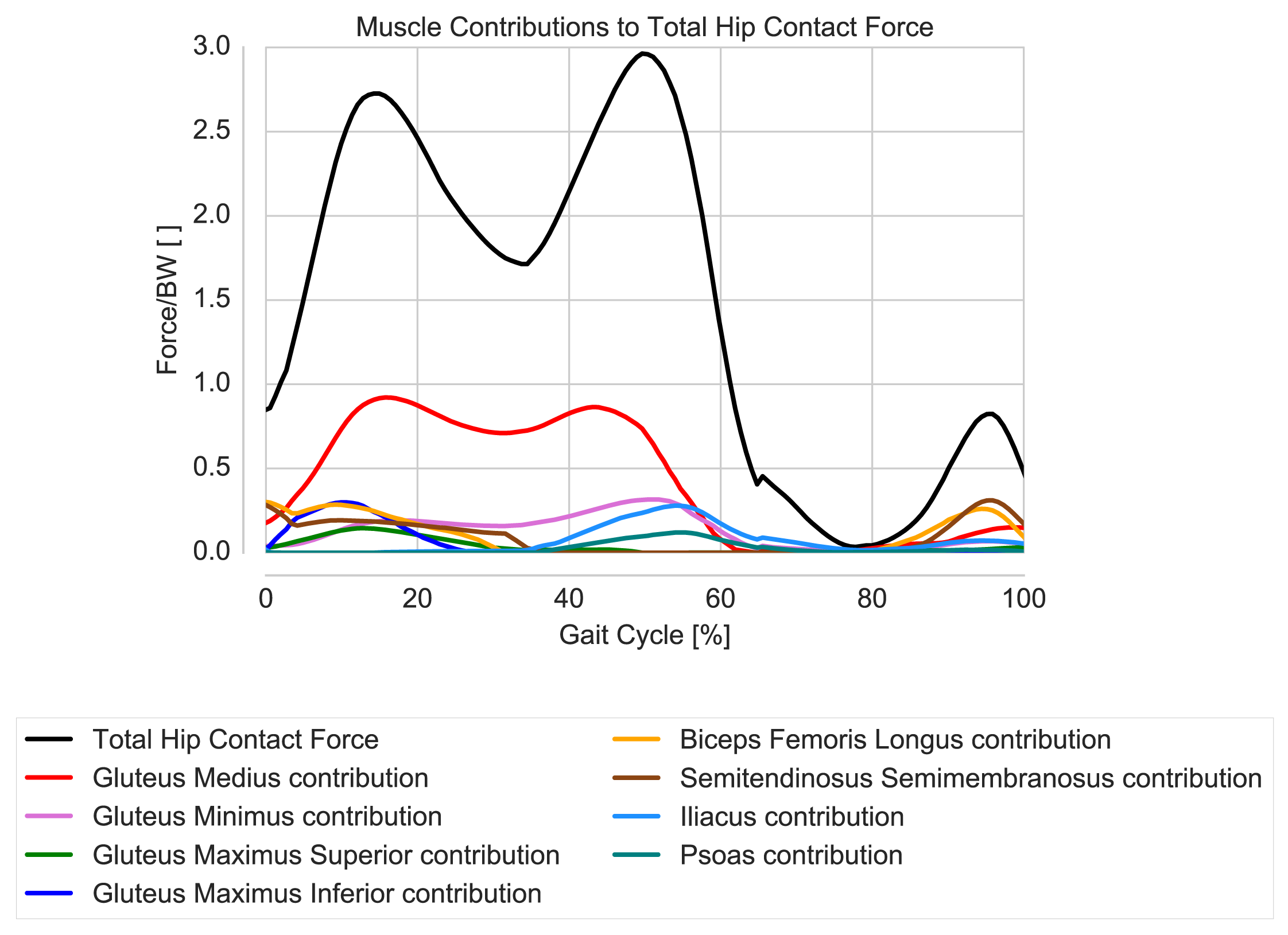

Supplement: S8 Fig — (TIF) [file pone.0204109.s008.tif]

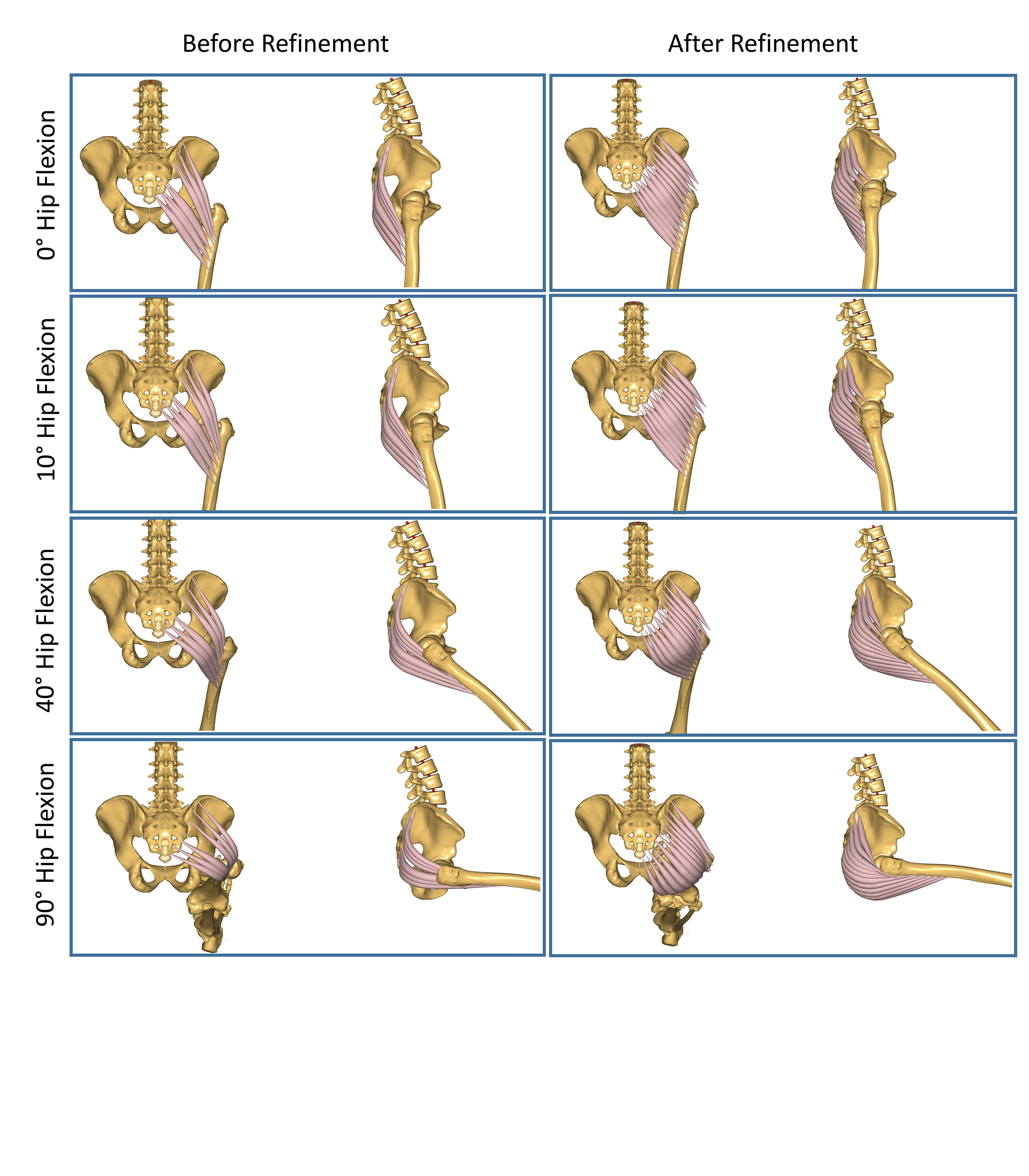

Supplement: S9 Fig — The Gluteus Maximus elements in the model are visualized from a posterior and a lateral view at different angles of hip flexion: 0°, 10°, 40°, and 90°. (TIF) [file pone.0204109.s009.tif]

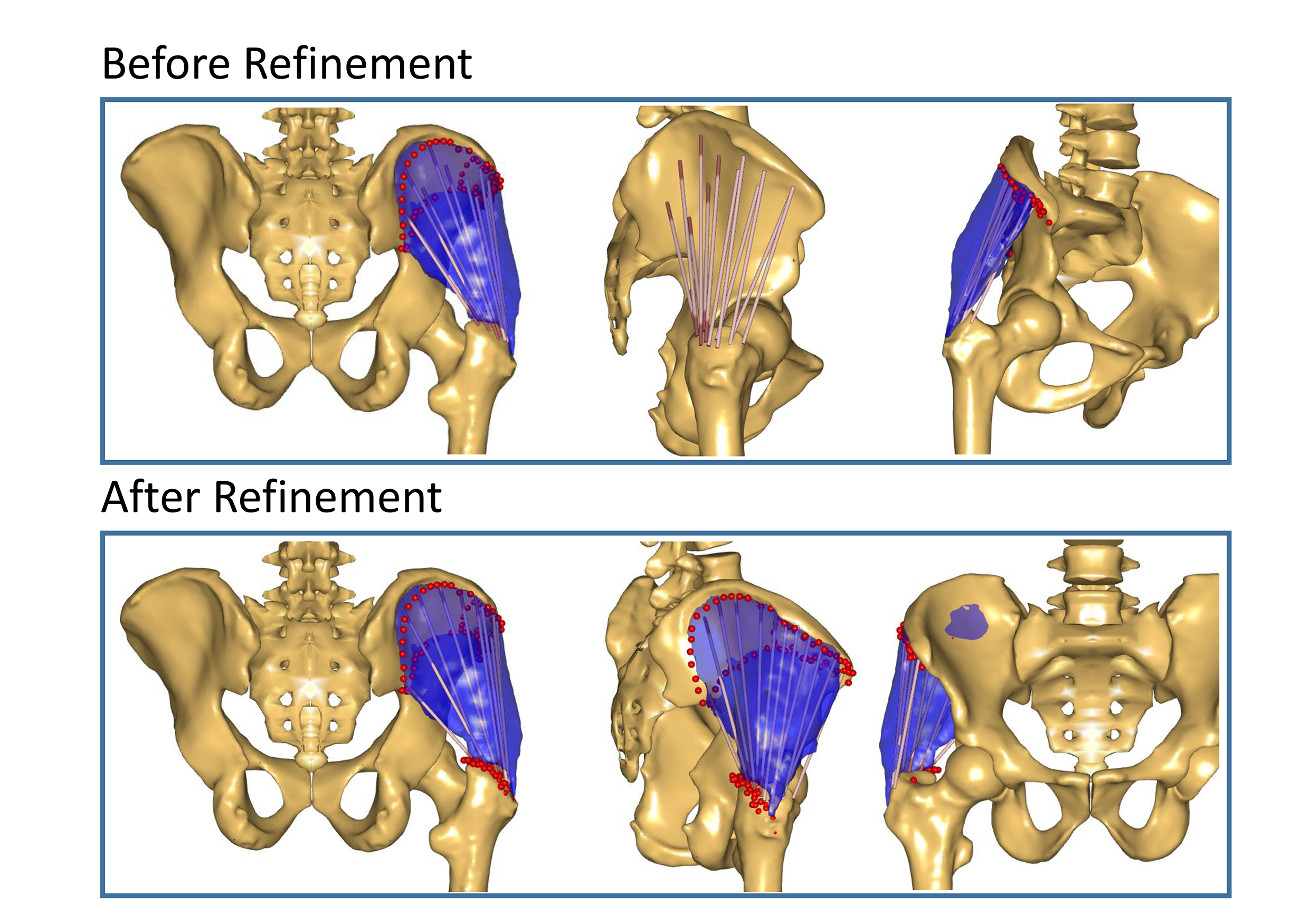

Supplement: S10 Fig — Gluteus Medius elements in the model are visualized in pink and overlapped with segmented muscle volumes (in blue) and contours of the origin and insertion areas (red dots) from the MRI scans. (TIF) [file pone.0204109.s010.tif]

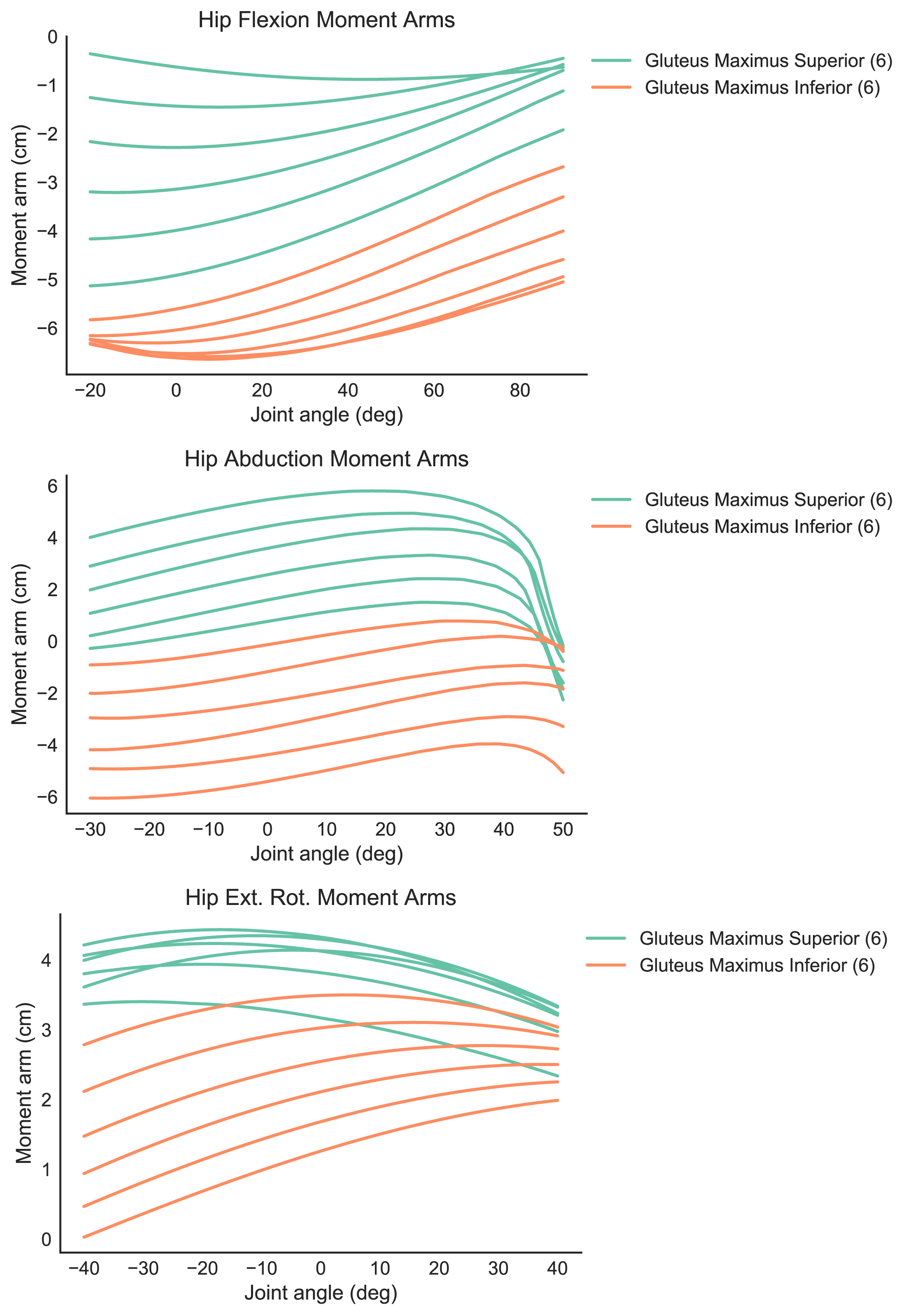

Supplement: S11 Fig — Moment arms are reported for the different elements of Gluteus Maximus over a range of hip flexion (+)/extension (-), abduction (+)/ adduction (-), and external (+)/ internal (-) rotation angles. In brackets the number of elements constituting the muscle is reported. (TIF) [file pone.0204109.s011.tif]

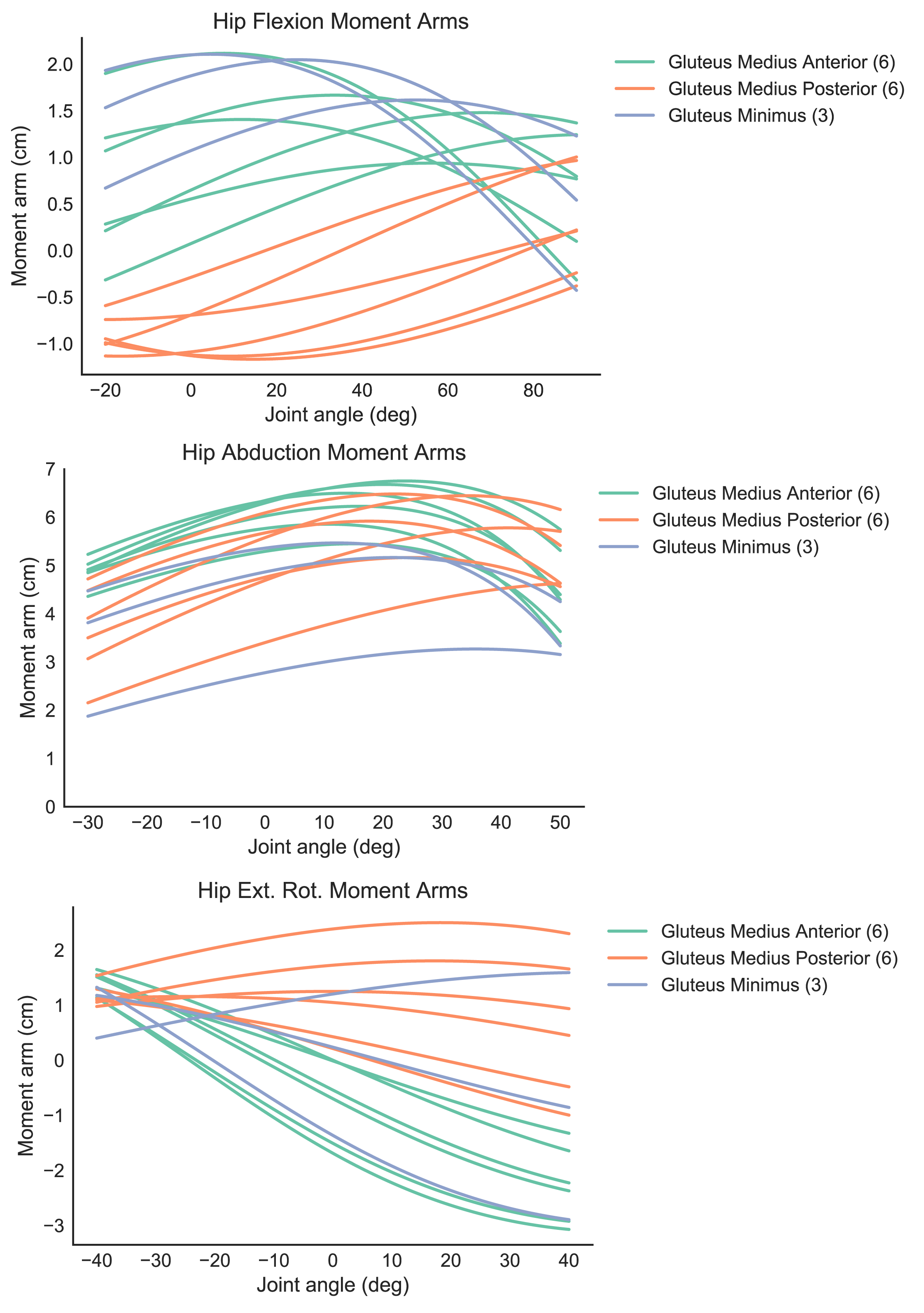

Supplement: S12 Fig — Moment arms are reported for the different elements of Gluteus Medius and Minimus over a range of hip flexion (+)/extension (-), abduction (+)/ adduction (-), and external (+)/ internal (-) rotation angles. In brackets the number of elements constituting the muscle is reported. (TIF) [file pone.0204109.s012.tif]

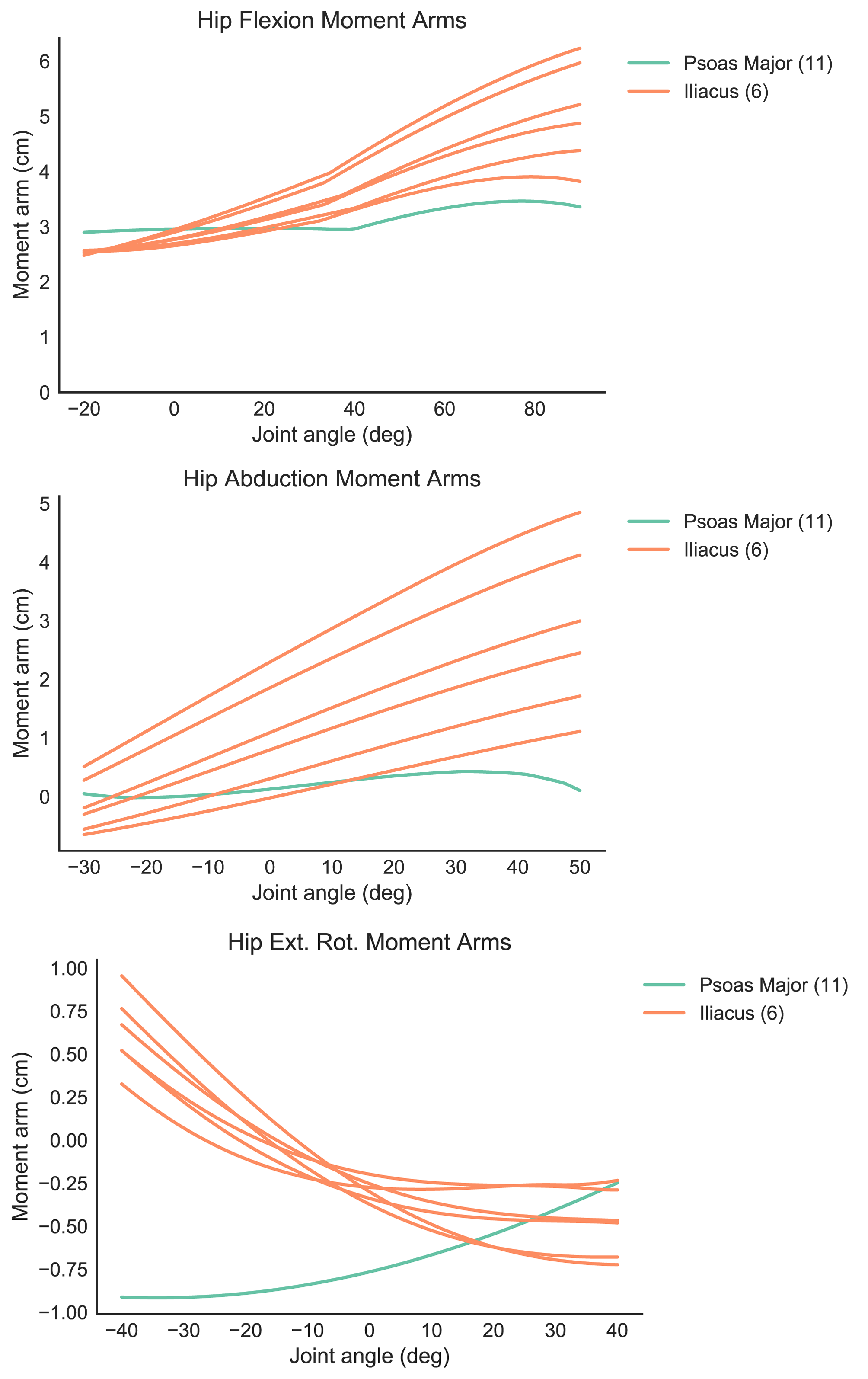

Supplement: S13 Fig — Moment arms are reported for the different elements of Iliacus and Psoas over a range of hip flexion (+)/extension (-), abduction (+)/ adduction (-), and external (+)/ internal (-) rotation angles. In brackets the number of elements constituting the muscle is reported. (TIF) [file pone.0204109.s013.tif]

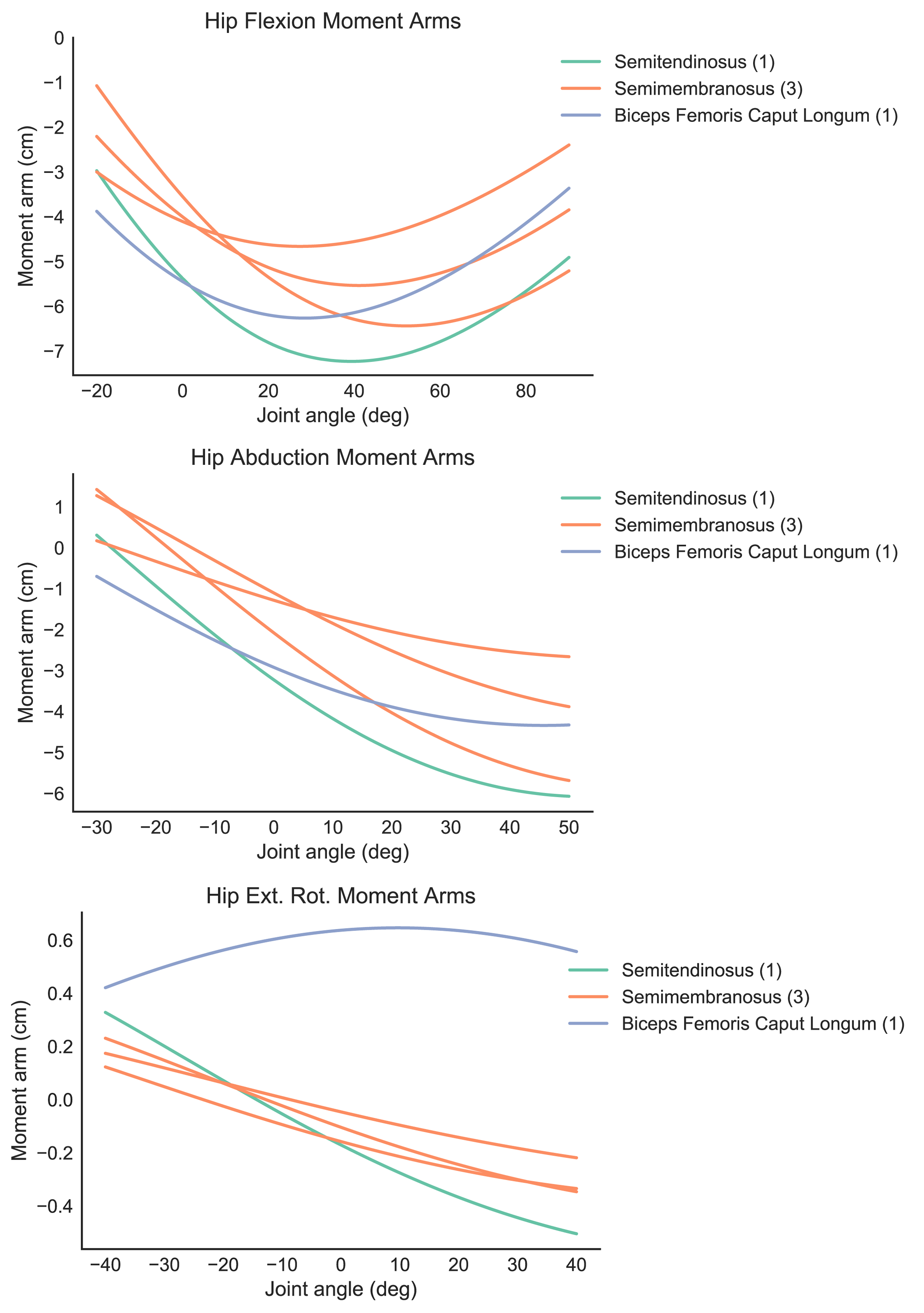

Supplement: S14 Fig — Moment arms are reported for the different elements of Semimembranosus, Semitendinosus, and Biceps Femoris over a range of hip flexion (+)/extension (-), abduction (+)/ adduction (-), and external (+)/ internal (-) rotation angles. In brackets the number of elements constituting the muscle is reported. (TIF) [file pone.0204109.s014.tif]

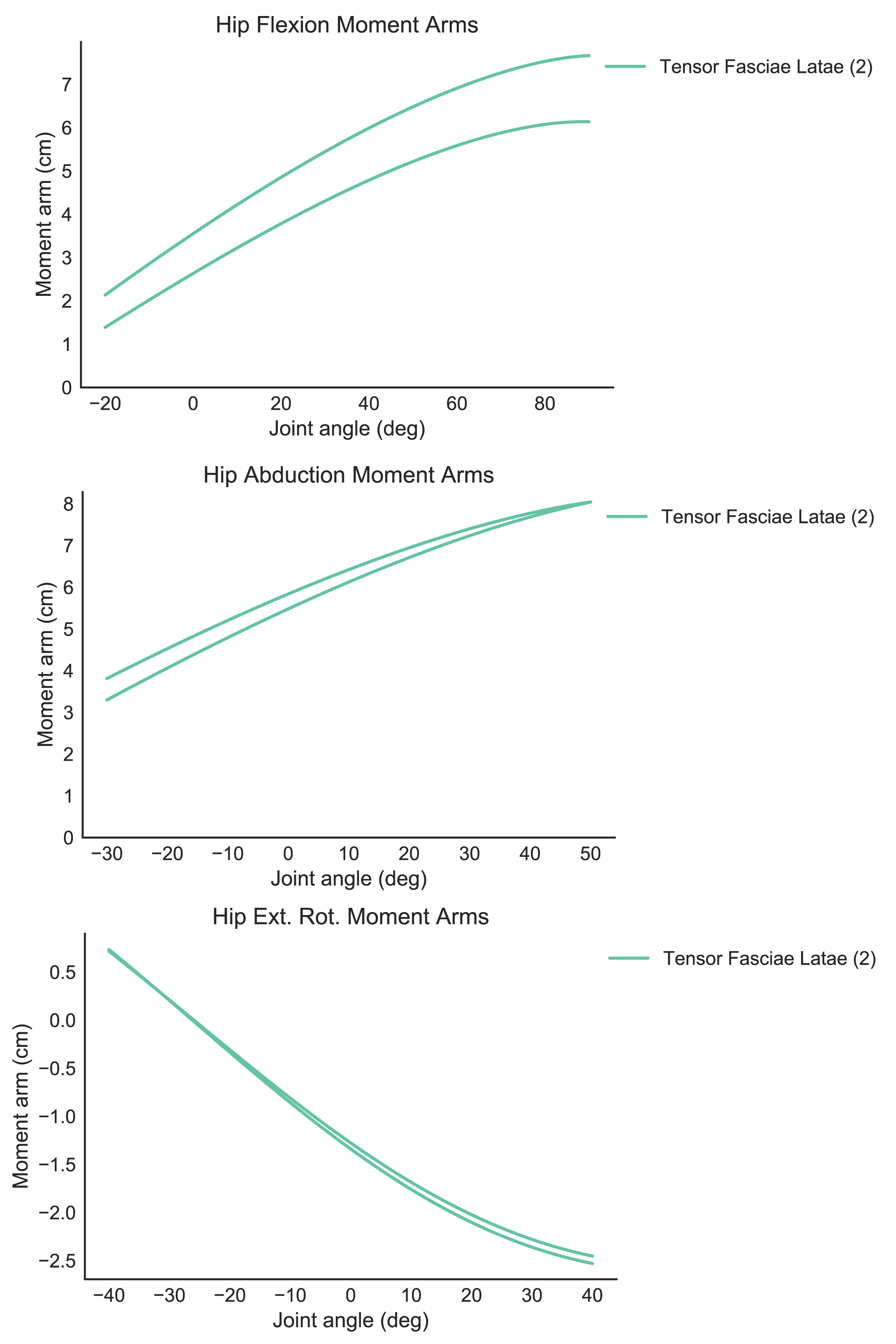

Supplement: S15 Fig — Moment arms are reported for the different elements of Tensor Fasciae Latae over a range of hip flexion (+)/extension (-), abduction (+)/ adduction (-), and external (+)/ internal (-) rotation angles. In brackets the number of elements constituting the muscle is reported. (TIF) [file pone.0204109.s015.tif]
